# Stilbenes from Vine Extracts: Therapeutic Potential and Mechanisms

**DOI:** 10.3390/ijms26178269

**Published:** 2025-08-26

**Authors:** Luís P. Brás, Ângelo Luís, Gregory Chatel, Sílvia Socorro, Ana Paula Duarte

**Affiliations:** 1 RISE-Health, Department of Medical Sciences, Faculty of Health Sciences, University of Beira Interior, Av. Infante D. Henrique, 6200-506 Covilhã, Portugal; luis.bras@ubi.pt (L.P.B.); angelo.luis@ubi.pt (Â.L.); ssocorro@fcsaude.ubi.pt (S.S.); 2 EDYTEM, CNRS, University Savoie Mont Blanc, F-73000 Chambéry, France

**Keywords:** waste viticulture, extraction, stilbenes, biological activities

## Abstract

The wine industry represents a significant economic sector; however, it generates large volumes of waste that can be valorized due to the presence of bioactive compounds, particularly stilbenes. These naturally occurring stilbenes exhibit remarkable potential in the prevention and treatment of various diseases, including cardioprotection, neuroprotection, antidiabetic properties, anti-inflammatory activity, and cancer prevention and therapy. This review discusses biosynthesis, structures, extraction methods, and mechanisms of action of stilbenes, with a particular emphasis on cancer prevention and treatment. Evidence from in vitro, in vivo, and clinical studies demonstrate that stilbenes modulate multiple molecular pathways by promoting apoptosis, inhibiting cell proliferation, and regulating inflammation, oxidative stress, and metabolism. However, the clinical application of stilbenes is limited by their low bioavailability. To overcome this, pharmaceutical formulations have been developed to enhance their stability and bioavailability, reduce side effects, and improve target interactions. These advances are expected to increase the therapeutic efficacy of stilbenes. Furthermore, information on the health benefits of less common stilbenes remains limited, highlighting the need for further research on these compounds.

## 1. Introduction

Viticulture is one of the most economically and culturally significant agricultural sectors worldwide. Wine is among the most widely produced and consumed beverages, with 94% of global production concentrated in just 29 countries [1]. Global consumption has reached approximately 221 million hectolitres, according to the International Organisation of Vine and Wine (OIV) [2]. Alongside this economic value, the wine industry generates large volumes of waste and byproducts estimated at 20–30% of the initial grape mass during winemaking [3,4].

These residues arise at two key stages: during harvesting (solid waste) and during processing (liquid waste) [5]. Solid waste includes grape pomace (45%), grape stalks (7.5%), grape pips (6%), grape marc (30%), and yeast (41.5%) [6,7,8]. Yeast lees alone account for ~5% of grape weight and contain ethanol, tartaric acid, phenolic compounds, and microbial biomass [9]. The fermentation and pressing steps contribute to an additional 20–25% of total waste [10].

In addition to winery waste, vineyard maintenance also contributes significantly to biomass generation. Annual pruning, essential for balanced plant development, produces an estimated 6–18 million tons of biomass each year, including shoots, branches, and leaves [11,12]. This accumulation results from vineyard renewal aimed at ensuring balanced growth. However, this material is often underutilized despite its potential for bio-based valorization. The biomass derived from vine waste and grape byproducts contains bioactive molecules important for human health [13].

While viticulture brings substantial economic benefit to many countries [14], the growth in grape production has led to increased vineyard surface area and value, along with a rise in the accumulation of both organic and inorganic waste [15,16]. This poses challenges to both economic and ecological management.

From an economic standpoint, inefficiencies persist, i.e., energy from renewable sources is poorly recycled, large quantities of waste are generated, and passive architectural strategies to optimize space and energy efficiency are underused [15,16,17]. Ecologically, the accumulation of waste increases the sector’s environmental footprint, which can be quantified using life cycle assessment (LCA) methodologies [18,19].

Despite these constraints, grapevines and their byproducts are rich in bioactive phytochemicals of potential value to the food, pharmaceutical, and cosmetics industries. These include dietary fiber, phenolics, proteins, and lipids [9,20,21,22,23,24,25,26], as well as stilbenes, which show promise as natural additives, antimicrobial and antioxidant agents, and even fillers in sustainable food packaging materials [15,27,28,29]. Leaves, in particular, contain organic acids, lipids, and polyphenols, and are already exploited in cosmetics [15,30]. Additionally, grapevine residues can be valorized as soil fertilizers or for bioenergy production [31].

The profile of polyphenols present in grape-based materials is subject to considerable fluctuation, reflecting not only genetic diversity and environmental variation, but also the interplay of cultivation and processing strategies. Each step from grape ripening to yeast selection shapes the ultimate spectrum of these compounds, with maceration time standing out for its impact on concentration [16,19]. Despite being a rich reservoir of bioactives like flavonols and anthocyanins, the grape skin’s direct contribution to wine composition is intentionally moderated; winemakers routinely remove these skins early to balance sensory qualities, inadvertently discarding a source of potential nutritional and therapeutic interest. This reality raises the question of how conventional practices might be optimized to both reserve product appeal and maximize the exploitation of health-promoting polyphenols, a challenge still largely unaddressed in mainstream oenology [28].

Grape seeds also contain high concentrations of antioxidants, including fiber, proteins, carbohydrates, lipids, minerals, phytosterols, phenolic compounds, vitamin E, and melatonin [15,32]. These polyphenols display activities relevant to stress response and chronic diseases. Notably, they intervene in cancer-related mechanisms such as tumor cell proliferation, metastasis, and drug resistance [33,34].

Among polyphenols, stilbenes are particularly interesting. As phytoalexins, they protect grapevines from pathogens and are mainly located in woody tissues like pruning canes and stalks [35]. The best known stilbene, trans-resveratrol (5-[(1E)-2-(4-hydroxyphenyl)ethenyl]-1,3-benzenediol; CAS: 501-36-0), has been extensively studied for its antioxidant, cardioprotective, and anticancer properties [36]. Stilbenes are ideal candidates for high-value valorization in the field of cancer prevention and anticancer therapy since they are found in pruning waste [15].

This review explores the therapeutic potential of vine-derived stilbenes particularly those found in pruning residues as anticancer agents. It covers their biosynthesis, extraction, chemical structures, biological activities, and mechanisms of action, while also addressing current limitations and future perspectives for their medical application.

## 2. Stilbenes from Grapevine: Biosynthesis, Structures, and Extraction

### 2.1. Polyphenols in Grapevine: General Classification and Biological Roles

Polyphenols, fundamentally, are the product of plant metabolism resulting in molecules that incorporate multiple phenolic motifs [37]. The chemical identity of a phenol, a compound consisting of an aromatic ring bearing at least one hydroxyl group, serves as the foundational structure for this diverse family. However, reducing these compounds to a structural formula overlooks the evolutionary rationale behind their biosynthesis, as well as their often-underestimated functional relevance in plant defense and, potentially, in human health [38].

The sheer heterogeneity of polyphenols, manifesting across grapevine tissues from root to leaf, defies easy classification. While chemists typically subdivide them by structure, highlighting families such as stilbenes or flavonoids, this approach captures only part of a dynamic and context-dependent reality [39,40]. Grape-derived plant material alone is home to a remarkable spectrum of compounds, containing not only well-characterized subclasses but also a long list of less-understood entities. This taxonomic richness, while scientifically intriguing, complicates efforts to standardize bioactive ingredient extraction for practical use [35].

Flavonoids are a class of polyphenols and are defined by a common structure composed of two aromatic rings joined by an oxygenated heterocyclic ring. Based on this core, they are subdivided into nine subclasses: chalcones, aurones, flavones, isoflavones, flavanols, anthocyanins, isoflavonoids, and bioflavonoids. Additionally, polyphenols can form associations with various carbohydrates and organic acids, contributing to their solubility, bioactivity, and interaction with other biomolecules [41].

Natural polyphenols function as secondary metabolites, enabling plants to respond to environmental stressors. These include ultraviolet (UV) radiation, pathogen attacks, nutrient-deficient soils, temperature extremes, drought conditions and herbivory [39,42,43]. As noted by Sies, polyphenol-rich plants and spices, especially those containing flavonoids, have been used for millennia in traditional Eastern medicine, although they are less common in Western therapeutic practices [44].

The extensive scientific literature now demonstrates that dietary polyphenols may confer health benefits across multiple biological systems. Their activities have been linked to protective effects against cancer, neurodegenerative diseases, cardiovascular dysfunctions, metabolic syndrome, diabetes, aging, and chronic inflammation [44,45,46,47,48,49,50,51,52,53,54,55,56,57,58,59,60,61,62,63].

### 2.2. Focus on Stilbenes: Structure, Diversity, Biosynthesis, and Natural Functions

Stilbenes are a class of polyphenols formed through the condensation of three C2 carbon residues with an activated hydroxycinnamic acid, a mechanism similar to that observed in flavonoid biosynthesis. Structurally, they consist of two aromatic rings linked by an ethylene bridge, forming the core diphenylethylene structure (C6-C2-C6) [64,65]. These compounds exist in both trans and cis isomeric forms, with the trans configuration being more stable and commonly found in nature [64,65,66].

According to the study by Goufo et al., stilbenes can be categorized based on polymerization degree, including monomers, dimers, trimers, tetramers, pentamers, and hexamers [35]. The most widely studied stilbenes such as resveratrol, pterostilbene, and piceatannol exhibit a range of biological properties including antioxidant, anti-inflammatory, and anticancer activities [67,68,69]. Pterostilbene additionally shows analgesic properties, while resveratrol has demonstrated protective effects against atherosclerosis [67,68].

**Figure 1:**
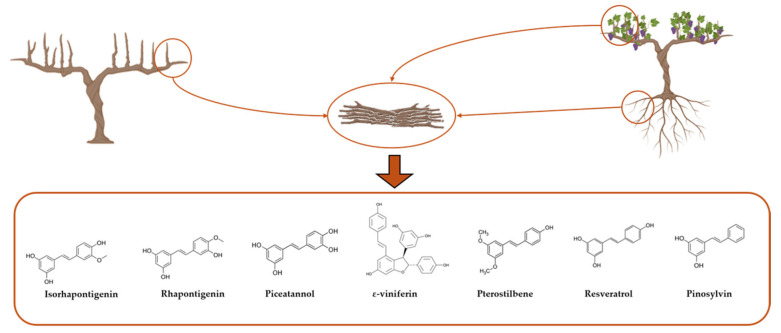
Schematic of the grapevine: stilbenes are primarily located in the wood, roots, branches, and stems, where they help prevent wood degradation and play defensive roles. These molecules exhibit structural variability, which determines their chemical behavior and bioactivity.

In grapevine, stilbene production is notably induced by mechanical damage, such as pruning of fresh grapevine canes, which stimulates the biosynthesis of compounds like resveratrol and piceatannol [70]. In a study by Guerrero et al., ε-viniferin was identified as the predominant stilbene (26–52%) in one-year-old grapevine canes, followed by other compounds such as trans-resveratrol, piceatannol, pinosylvin, rhapontigenin, pterostilbene, and isorhapontigenin, whose levels varied over time (Figure 1) [71]. These molecules vary structurally, and this variation determines their chemical behavior and bioactivity. For instance, hydroxylated and glycosylated stilbenes have improved water solubility, enhancing incorporation into hydrophilic systems. Methoxylated stilbenes, by contrast, are more lipophilic and bioavailable. Glycosylated forms tend to be less stable and more prone to isomerization than their aglycone counterparts. Ortho-hydroxylated stilbenes, due to the ability to form stable semiquinone radicals, display greater antioxidant, anti-inflammatory, and anticancer effects compared to meta-hydroxylated isomers [72].

Stilbenes occupy a dual role within the plant kingdom, acting both as an ever-present line of defense and as rapid responders to environmental threat. Their involvement in shielding plants from infectious agents or harsh climatic conditions illustrates the evolutionary value of chemical versatility [71,73,74,75]. Not uniquely confined to grapevines, these compounds are distributed across a wide botanical landscape, from staple crops to regional medicinal herbs. The notable presence of pterostilbene in berries and residues underlines the untapped reservoir of such molecules in both dietary and agricultural contexts. This cross-species distribution implicates stilbenes as a convergent solution to common biological challenges but also complicates efforts to establish source-specific therapeutic claims [71,76].

**Figure 2:**
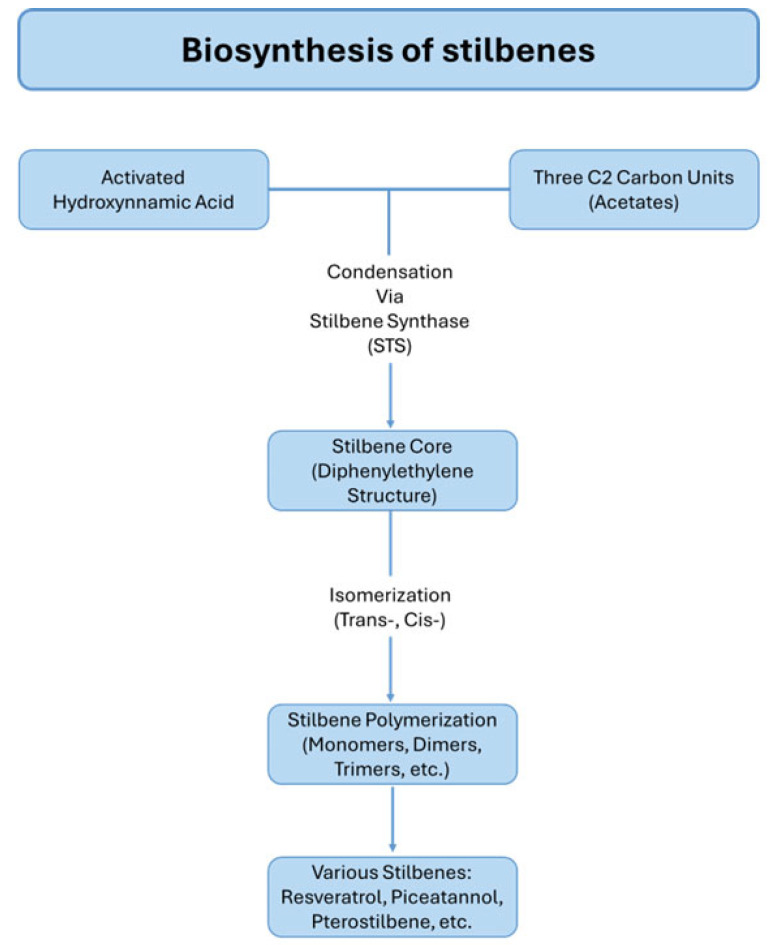
Flow diagram of stilbenes’ biosynthesis.

The biosynthesis of stilbenes is catalyzed by stilbene synthase (STS), an enzyme that evolved from chalcone synthases (CHS) through convergent evolution. This enzyme family displays tissue-specific and development-specific expression patterns. For example, STS gene expression is lower in young grapevine leaves but increases significantly in mature leaves and grape skins during ripening especially in varieties such as Cabernet Sauvignon and Norton peaking at harvest time (Figure 2) [73,77].

Grapevine byproducts like pomace and winemaking residues are rich in polyphenols, including stilbenes [78]. In viticultural waste, total stilbene concentrations range from 2400 to 5800 mg/kg dry weight (DW) [71]. Cebrián et al. observed the accumulation of phenolic compounds in grapevine buds after 1, 3, and 6 months of post-pruning storage in two Vitis vinifera varieties [79]. In grapevine canes, trans-resveratrol levels range from 441 to 7532 mg/kg DW, while trans-ε-viniferin concentrations vary from 1218 to 5341 mg/kg DW, depending on cultivar, vintage, and storage conditions.

Dormancy in grapevine, including endodormancy and ecodormancy, which occur during cold climates, significantly influences the biochemical composition of canes [80]. Hybrid cultivars adapted to northern latitudes, such as those grown in Estonia, have been found to accumulate high levels of dietary stilbenes like resveratrol and viniferin [81,82].

Grapevine canes, rich in bioactive stilbenes, demonstrate notable antifungal and antioxidant properties, making them valuable for nutraceutical applications [71]. Bud extracts contain up to 29% stilbenes and their incorporation in winemaking presents an eco-friendly strategy to reduce sulfur dioxide (SO_2_) use, particularly in white wines [83]. Toasted grapevine canes also enhance the antioxidant properties of wine by contributing high levels of prodelphinidins and stilbenes [84].

Furthermore, grapevine leaves and cane extracts are a source of bioactive antioxidant molecules and other valuable metabolites for pharmaceutical, nutraceutical, and food applications [81,85]. The abundance of raw materials from viticultural waste provides a compelling opportunity for industrial valorization, given the commercial and pharmacological potential of stilbenes [86,87].

### 2.3. Extraction Methods of Polyphenols and Stilbenes

The pursuit of efficient polyphenol extraction continues to evolve, reflecting the chemical diversity and sensitivity of these compounds. While early protocols relied on harsh conditions and extended processing times, contemporary approaches are trending toward gentler and rapid techniques, such as microwave-assisted extraction (MAE), ultrasound-assisted extraction (UAE), and ultrahigh-pressure extraction, which preserve and selectively recover targets from complex matrices. A critical, though often underestimated, factor in yield and purity is the nature of the solvent system that balances polarity to suit the solubility profile of the molecules of interest. Complexities such as acid–base stability and matrix interference persist, challenging researchers to continually fine-tune protocols for industrial and pharmacological scalability [88].

Conventional methods for extracting stilbenes particularly trans-resveratrol and trans-ε-viniferin from grape canes primarily rely on conventional solid–liquid extraction (CSLE). This involves maceration in a solvent, with or without stirring, under atmospheric pressure and temperatures ranging from ambient to reflux conditions. Extraction efficiency is influenced by multiple factors, including the solvent type, solvent-to-solid ratio, temperature, extraction time, pH, and light exposure. Hydroalcoholic mixtures such as ethanol/water are commonly used due to their efficacy and non-toxicity [89]. Angel et al. showed that ethanol/water mixtures outperform other solvents in yielding stilbenes [90,91]. An increase in the solvent-to-solid ratio generally improves yield, with optimal ratios typically between 5 and 50 mL/g. Higher temperatures also enhance extraction, with optimal results achieved between 20 °C and 80 °C without degrading the compounds. Prolonged extraction times may increase concentration but can also cause compound degradation. Additionally, alkaline conditions and UV light exposure negatively affect stilbene stability.

Emerging techniques such as UAE, MAE, and pressurized solvent extraction (PSE) offer several advantages over conventional methods. UAE employs acoustic cavitation generated by ultrasonic waves to enhance mass transfer, disrupt plant tissues, and increase surface area, thus improving yields and reducing extraction time and energy use [92]. Despite its benefits, UAE requires careful optimization of parameters like frequency, power, and temperature. MAE, on the other hand, uses microwave energy to heat solvents, promoting efficient cell disruption and reducing extraction times. This method enables uniform heating, rapid energy transfer, and lower solvent consumption. Compared to conventional heating, MAE reduces energy waste and enhances extraction of bioactive molecules in an environmentally friendly manner [92,93,94,95,96]. Optimized MAE conditions have been shown to increase stilbene recovery, though comparisons across studies are often difficult due to variability in extraction parameters [33,89]. PSE employs pressures between 10 and 15 MPa to maintain solvents in a liquid state above their boiling points, improving solubility and mass transfer. For stilbenes, ethanol/water (25:75, *v*/*v*) at a 30 mL/g ratio, 105 °C, 1 mL/minute flow rate, and 5.2 MPa pressure has demonstrated higher diffusivity and shorter extraction times compared to conventional methods. One study reduced extraction time from 8 h (Soxhlet) to 10 min using PSE, with slightly increased yields [89].

A key trend in extraction science is the shift towards processes that minimize time and energy consumption without compromising yield. As demonstrated by recent comparative studies, techniques leveraging microwave or pressure are especially effective, and may even obviate the need for added solvents when the inherent moisture of the sample is sufficient. Despite universal gains in speed and ecological footprint, not all advanced workflows are equally user-friendly; fine-tuning parameters, particularly for ultrasound-based approaches, remain a nuanced, trial-and-error exercise. This reality tempers the initial enthusiasm for high-tech solutions with the need for context-sensitive optimization in practical settings [36].

Extraction yields are affected by grapevine variety and environmental factors such as UV exposure, soil type, and vineyard practices like leaf removal or fertilization. Even within the same variety, yields may vary due to biological and climatic influences. Post-pruning parameters such as storage duration, cane provenance, and physiological stage also play a significant role in extraction optimization [89].

Trans-resveratrol and trans-ε-viniferin are the dominant stilbenes in grape canes, but other oligomeric stilbenes such as hopeaphenol, ampelopsins, vitisins, pterostilbene, piceatannol, and polydatin are also present and bioactive [97,98,99]. Nonetheless, stilbenes account for only 3.1% of the dry mass of grape canes, meaning over 95% of the biomass remains underutilized after extraction [89].

## 3. Biological Activities of Stilbenes and Health Implications

### 3.1. Bioavailability and Metabolism of Stilbenes

Despite their reputation for limited absorption, some stilbenes and their derivatives have been documented to attain concentrations in vivo sufficient for biological relevance. The extent to which this translates into tangible physiological effects, however, is still fiercely debated, and often contingent on the nuances of both compound structure and formulation [100]. However, the most researched stilbene, resveratrol, has a short half-life because to its quick metabolism (14 min), limited oral bioavailability (20–30%), and low water solubility (<0.05 mg/mL) [101,102]. Oral absorption in humans is around 75%, but extensive intestinal and hepatic first-pass metabolism (mainly glucuronidation and sulfation) reduces systemic availability [103]. Stilbenes’ complexation with cyclodextrins improves the phenolic compounds’ solubility but not their bioavailability within target tissues [104]. However, other formulation strategies such as incorporation into bile acid complexes, liposomes, nanoparticles, or co-administration with absorption enhancers like piperine can significantly increase plasma concentrations and tissue delivery [105,106,107,108,109,110].

Pterostilbene exhibits superior oral bioavailability (~80%), attributed to its increased lipophilicity (due to two methoxy groups) and higher metabolic stability (one free hydroxyl), resulting in slower glucuronidation and a longer half-life compared to resveratrol (1.73 vs. 1.48 h) [111,112,113,114,115,116]. Gnetol possesses lower bioavailability (~6.6%) but a longer half-life (4.2 h) and sustains serum levels of its glucuronide metabolite, likely supporting pharmacological activity [117,118]. In contrast, ε-viniferin and other stilbenes (piceatannol, pinostilbene) display very low oral bioavailability (<1%, or 0.8% for ε-viniferin), attributed to poor absorption and/or extensive metabolism [119].

Stilbenes are mainly metabolized via phase II reactions (glucuronidation, sulfation) at phenolic groups [120,121,122,123,124,125,126,127]. In human plasma, phase II conjugates predominate (resveratrol-3-sulfate being the most abundant), whereas in animal models, glucuronide forms are often higher. Some metabolites, especially conjugated forms, may serve as reservoirs, being deconjugated to release active stilbene, and enterohepatic recirculation has been observed. Dihydro-resveratrol, formed by intestinal hydrogenation, retains relevant biological activity, particularly in the colon [128,129].

Tissue distribution studies show that pterostilbene and its sulfate accumulate in tissues, particularly in the central nervous system, unlike resveratrol, whose plasma and tissue levels decrease quickly. Piceatannol and pinostilbene are efficiently glucuronidated and show high hepatic concentrations but low systemic exposure and rapid clearance [130,131,132,133,134]. Piceatannol can also serve as a metabolic product of resveratrol via CYP1B1, suggesting a prodrug relationship [135].

Nanotechnological strategies including PEGylated liposomes and lipid nanocapsules can significantly enhance brain delivery and systemic concentrations of stilbenes, addressing issues of chemical instability and photosensitivity [78,105] and novel soluble galenic formulations also markedly increase resveratrol’s bioavailability and biological effects. In clinical settings, stilbenes are generally well tolerated even at high doses; adverse effects are mild and mostly gastrointestinal, and doses above 1 g/day are avoided due to tolerability limits [136,137,138,139,140].

Metabolite profiles are complex, including a wide array of glucuronide and sulfate forms, as well as hydrogenated and methylated derivatives [120,121,122,123,124,125,126,127,133]. For some stilbenes, tissue- or adipose-localized conjugates may be reconverted in situ, maintaining local activity. Excretion occurs mainly via feces rather than urine, as seen for ε-viniferin. Detection of certain stilbenes (e.g., ε-viniferin) in rat brain tissue indicates the ability to cross the blood–brain barrier, suggesting potential for central nervous system effects [141,142].

In summary, stilbenes demonstrate varied pharmacokinetic profiles, mainly limited by low bioavailability and rapid metabolism. Pterostilbene stands out for its higher oral bioavailability, tissue accumulation, and longer half-life, whereas other stilbenes may benefit from novel delivery systems to enhance clinical efficacy and expand therapeutic potential.

### 3.2. Antioxidant and Anti-Inflammatory Properties

**Figure 3:**
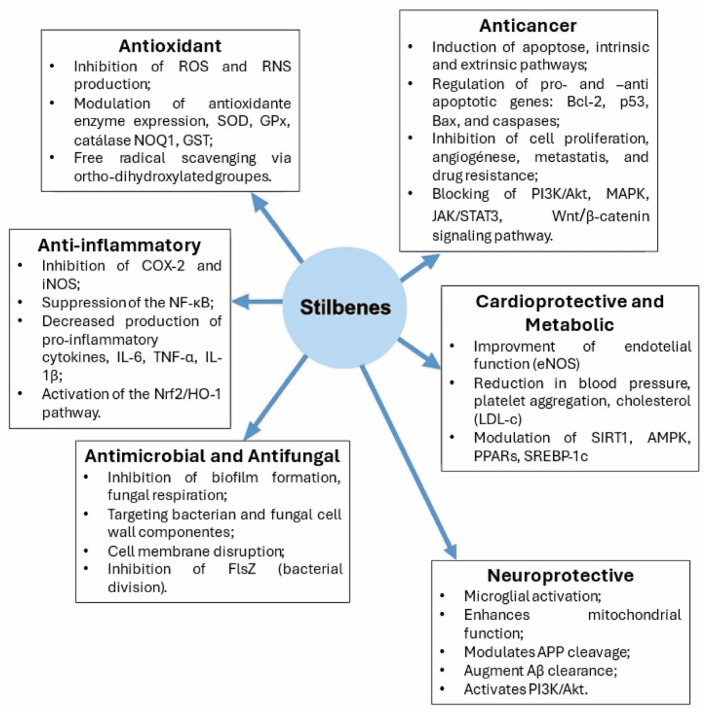
Diagram of mechanisms of biological activities of stilbenes.

The preventive effects of stilbenes on various diseases are largely attributed to their antioxidant activities, including anti-cyclooxygenase action and modulation of lipid and lipoprotein metabolism (Figure 3) [77]. Stilbenes counter oxidative stress by inhibiting reactive oxygen and nitrogen species (ROS and RNS), a property influenced by hydroxyl group position and number. Ortho-dihydroxylated compounds, like piceatannol, are more potent due to enhanced stabilization of semiquinone radicals, particularly at the R4’s position of resveratrol. Their antioxidant activity is also modulated by pH and protonation states. While direct radical scavenging is limited in vivo, stilbenes regulate antioxidant enzymes such as catalase, superoxide dimustase (SOD), nicotinamide adenine dinucleotide phosphate (NADPH) oxidase, glutathione peroxidase (GPx), glutathione S-transferase (GST), and NQO. For instance, resveratrol upregulates catalase, GPx1, SOD1, and SOD3, while suppressing NOX2 and NOX4 in ApoE-KO mice [82].

Stilbenes also exert strong anti-inflammatory effects. They inhibit cyclooxygenase (COX) enzymes and nitric oxide synthases, reduce cytokine production, and suppress nuclear factor κB (NF-κB) signaling. Stilbenes such as piceatannol, pterostilbene, pinosylvin, desoxyrhapontigenin, and rhapontigenin inhibit inducible nitric oxide synthase (iNOS), reduce prostaglandin synthesis, and COX-2. Oxyresveratrol and piceatannol also inhibit nitrite production and iNOS, and resveratrol, oxyresveratrol, and piceatannol block NF-κB activation. Pterostilbene inhibits nuclear translocation of NF-κB and reduces pro-inflammatory cytokine production. Piceatannol is more effective than resveratrol in inhibiting COX-2, NF-κB activation, and cytokine production, while pinosylvin inhibits COX-2 with nearly twice the potency of resveratrol. Rhapontigenin is less active than desoxyrhapontigenin due to a missing hydroxyl at R3’ [82].

Piceatannol may contribute to anti-inflammatory responses by activating pathways involving heme oxygenase-1 (HO-1). In human endothelial cells, it upregulates HO-1 expression by activating the nuclear factor erythroid 2-related factor 2 (Nrf2). Inhibition of HO-1 negates piceatannol’s effects, restoring the expression of tumor necrosis factor-α (TNF-α), interleukins-6 (IL-6), and NF-κB and p65 phosphorylation [143]. Piceatannol has also shown the ability to increase HO-1 expression in macrophages, endothelial cells, mammary epithelial cells, rat liver, and neuronal cells [144,145,146,147,148,149]. However, it is worth noting that HO-1’s role in metabolic disorders is not well understood and, in some cases, has shown pro-inflammatory effects [150]. Thus, it is possible that piceatannol’s anti-inflammatory effects are mediated through the regulation of the upstream activator of HO-1, Nrf2 [147].

Several studies have demonstrated piceatannol’s ability to inhibit inflammatory pathways. Free fatty acids (FFAs) promote inflammation, with palmitic acid having pro-inflammatory properties. Piceatannol prevents the inhibitory effects of palmitic acid on insulin receptor substrate 1 (IRS-1) phosphorylation, glucose uptake, eNOS phosphorylation, and nitric oxide production in human endothelial cells [143]. Furthermore, piceatannol reduces lipopolysaccharide (LPS)-induced protein expression of IL-6 and TNF-α, attenuates NF-κB and signal transducer and activator of transcription 3 (STAT3) signaling in macrophages, and prevents IL-6 secretion and STAT3 and STAT5 signaling in human lymphocytes [144,151,152,153].

In addition, resveratrol enhances tetrahydrobiopterin (BH4) synthesis in cell and animal models, showing reduced oxidation [154]. BH4 is reduced by oxidative stress, negatively affecting various physiological functions [155]. Resveratrol can inhibit cyclic adenosine monophosphate (cAMP) phosphodiesterase, thereby increasing cellular levels of cAMP in cell and animal models [156]. Phosphodiesterases (PDEs) are enzymes that degrade cAMP and cyclic guanosine monophosphate (cGMP), which are second messengers involved in regulating numerous genes and cellular functions [157].

Stilbenes, particularly resveratrol, can activate cAMP signaling pathways. For in-stance, resveratrol boosts antioxidant defenses in aging cells, where cAMP induces Nrf2 expression. This pathway is also activated by alpha-melanocyte-stimulating hormone, a hormonal activator of melanocortin receptors coupled to the G-protein–cAMP cascade [158]. Protein kinase A and cAMP response element-binding protein (CREB) mediate Nrf2 activation through cAMP response elements (CREs) biding in their promoters, resulting in Nrf2 and Nrf2-related gene (e.g., glutathione S-transferase pi 1 and NOS) transactivation [159,160,161].

One of the more intriguing avenues in stilbene pharmacology concerns the interplay between resveratrol and energy-sensing molecular machinery. By interfering with phosphodiesterases, resveratrol alters intracellular calcium and thereby activates a cascade that converges on Adenosine monophosphate-activated protein kinase (AMPK), a critical metabolic checkpoint [162]. These events ripple outward, promoting shifts in both NAD^+^ pools and sirtuin activity, which could, in theory, recalibrate key metabolic regulators implicated in chronic disease. Yet, a challenge persists, i.e., drawing causal links from these signatures in model systems back to consistent outcomes in whole organisms remains elusive [163,164,165].

### 3.3. Antimicrobial and Antifungal Activities

Stilbenes have demonstrated antibacterial, antiviral, and antifungal activity (Figure 3) [105,166,167,168]. Resveratrol and its analogues act as inhibitors of fungal tyrosinase [106,107]. In addition, stilbenes inhibit fungal cellular respiration and the lipid peroxidation of fungal membranes [136]. The antifungal activity of resveratrol inhibits a broad spectrum of fungi, including *Pyricularia oryzae*, *Plasmopara viticola*, *Cladosporium cucumerinum*, *Sphaeropsis sapinea*, *Phytophthora capsici*, *Phytophthora colocasiae*, *Botrytis cinerea*, *Candida albicans*, and *Colletotrichum gloeosporioides* [137,138,139,169]. Pinosylvin shows the highest efficacy against Gram-negative bacteria, followed by resveratrol, piceatannol, oxyresveratrol, and pterostilbene. Against Gram-positive bacteria, pinosylvin and pterostilbene are more effective than resveratrol. Pterostilbene’s hydrophobicity and methylation improve membrane penetration and antifungal potency up to five times higher than resveratrol in inhibiting conidial germination and in vitro mycelial growth [72].

The first mechanism for antimicrobial activities involves their ability to inhibit conventional targets, such as the cell’s membrane and wall, cell division, DNA, mitochondria, the calmodulin–calcineurin pathway, and the phosphoenolpyruvate (PEP)-dependent phosphotransferase system. The second mechanism relates to their ability to act as antibiofilm and antivirulence agents. Thus, stilbenes inhibit biofilm formation and assist in the eradication of already formed biofilms. Furthermore, another mechanism of stilbenes is their ability to reverse drug resistance through inhibition of alternative targets, target-modifying enzymes, and antibiotic-modifying enzymes [170]. Stilbenes have an antimicrobial mechanism that involves disrupting bacterial membranes, resulting in the leakage of cellular contents and leading to cell death. Pterostilbene, resveratrol, and toremifene (an anticancer agent approved by the Food and Drug Administration (FDA) and synthesized in 1981) have demonstrated antimicrobial activity through cell membrane disruption [171,172,173]. The membrane-associated protein phosphatidylglycerophosphate synthase (PgsA) catalyzes the substitution of cytidine monophosphate with glycerol phosphate to produce phosphatidylglycerol phosphate (PG-P). Subsequently, PG-P is dephosphorylated by PgpP to yield phosphatidylglycerol (PG), which is one of the main components of the cell membrane [174].

Another mechanism involves the action on the bacterial cell wall, which consists of a complex peptidoglycan polymer net composed of N-acetylmuramic acid and N-acetylglucosamine, with an attached pentapeptide. The biosynthesis of bacterial peptidoglycan is catalyzed by sequential Muramyl ligases in the intracellular steps. Stilbenes have the ability to inhibit MurD, an enzyme that catalyzes the reaction from UDP- N-acetylmuramic-Ala to UDP- N-acetylglucosamine-dipeptide [175].

Wall teichoic acids are anionic glycopolymers anchored in the cell walls of Gram-positive bacteria that play critical roles in bacterial physiology. Wall teichoic acids’ biosynthesis initiates with a reaction is catalyzed by the membrane-associated glycosyltransferase TagA [111]. Stilbenes have shown promising activity against Gram-positive bacteria by targeting cell wall teichoic acids [111].

The fungal cell wall is composed of complex components, mainly polysaccharides (chitins, glucans, and mannans), lipids, and proteins. Stilbenes inhibit the activity of chitin synthases [176]. NADPH–cytochrome P450 reductase chemokine receptor (CCR1) is involved in the yeast cell wall. Stilbenes have the ability to inhibit the activity of NADPH–cytochrome P450 reductase CCR1, leading to defects in yeast cell wall integrity [177].

Stilbenes have shown highly promising results in reducing the mycelial growth of phytopathogenic fungi such as *Pythium aphanidermatum*, *Rhizoctonia solani Kuhn*, *Exserohilum turcicum*, and *Fusarium oxysporum*, as well as the yeast *Saccharomyces cerevisiae*, in both agar medium and broth microdilution assays, using chlorothalonil as a positive control [178]. A series of resveratrol oligomers were purified from the canes of Vitis vinifera. These compounds were tested against *Candida albicans* [179,180]. Pterostilbene was shown to be a more potent inhibitor compared to 5-fluorocytosine, a reference antifungal agent used against *Candida albicans*. Moreover, pterostilbene proved to be a promising inhibitor against 12 non-albicans *Candida* (NAC) species, including emerging pathogens such as *Candida guilliermondii* and *Candida famata*. These findings support the idea that naturally occurring stilbene-type phytoalexins, produced by plants and certain bacterial species, serve as defense mechanisms against pathogens and represent a rich source for the discovery of novel fungicides [181].

Stilbenes can inhibit DNA synthesis. For example, trans-dihydroresveratrol dimer has been shown to block the ATP-binding site of DNA gyrase, inhibiting, therefore, the enzyme’s activity and consequent DNA synthesis [182]. Another mechanism is causing DNA damage. For instance, oxyresveratrol binds directly to DNA, inducing the molecules’ cleavage and subsequent mitochondria-mediated apoptosis in *Candida albicans* [183]. Resveratrol’s prooxidant activity induces DNA damage through an increase in ROS, malondialdehyde accumulation, and glutathione depletion in *Salmonella typhimurium* [184].

Beyond their central role in cellular energetics, mitochondria stand at the crossroads of survival and programmed cell death. Loss of membrane integrity precipitates a sequence that leads inevitably to apoptosis [185]. Evidence implicates resveratrol as a potent modulator of this process, which is able to destabilize mitochondrial function and tip the scales towards cell death, as seen in both pathogen and tumor models. This mechanistic insight, while compelling, raises further questions about tissue selectivity and the balance between therapeutic efficacy and toxicity [186].

The cell membrane and wall, DNA, and mitochondria are common targets in antimicrobial therapies and, therefore, stilbenes’ effects on the mentioned cellular structures are of interest when designing new treatment strategies for pathologies caused by microorganisms. For example, resveratrol and piceatannol perform a reversible inhibition in ATPase activity and consequent ATP synthesis in *Escherichia coli* [187]. Moreover, resveratrol’s antibacterial activity extends to the downregulation of FtsZ expression and inhibition of Z-ring formation, a dynamic structure essential to cell division in prokaryotic organisms since it recruits division-related proteins and directs septal peptidoglycan synthesis [188,189].

Conventional antibiotics are becoming less effective overtime due to an increase in the number of multidrug-resistant microorganisms. Virulence and its associated factors determine pathogenicity and allow pathogens to acquire desired characteristics, such as immune evasion or modulation, colonization, and tissue damage. Toxins are also implicated in pathogenesis and include superantigens, surface proteins, hemolysins, and leukocidins. The pathological mechanism of α-hemolysin (Hla) secreted by *Staphylococcus aureus*, for example, relies on the direct binding to erythrocytes’ cell membrane and subsequent pore formation and lysis. Thus, resveratrol and trans-stilbene have the ability to inhibit hemolysis caused by *Staphylococcus aureus* through the repression of the *hla* gene expression, thereby reducing the microorganism’s virulence [140]. Furthermore, the two-component system SaeRS exerts essential functions in virulence factors’ production [108]. Resveratrol can target the mentioned system, resulting in the downregulation of *saeRS* and consequent reduction in α-hemolysin secretion [109].

It is estimated that between 40% and 80% of the existing bacteria and archaea reside in biofilms, a macrocolony of microorganisms attached to a surface that constitutes a contributing factor in the development of chronic infections [110,112,113]. Biofilms offer microorganisms an advantage under various environmental challenges [114].

Biofilm formation occurs in five progressive stages, which include initial reversible attachment, irreversible attachment, first layer formation, mushroom- or tower-shaped structure formation, and dispersion and reattachment [112]. Stilbenes are capable of disturbing the hallmarks in biofilm formation, arresting their maturation, and allowing for the elimination of the mentioned structure. For example, resveratrol inhibits the production of flagellin and, consequently, reduces the motility of *Proteus mirabilis*, thereby preventing swarmer cell invasion into human uroepithelial cells [115]. Resveratrol also impairs the motility and adhesion of *Salmonella typhimurium* to HeLa cells through downregulation of flagellar genes [190]. Furthermore, resveratrol suppresses *fimA* and *xadA* expression, resulting in low levels of afimbrial adhesin and impaired adhesion capacity of *Xylella fastidiosa* to host cell surfaces [191]. The downregulation of fimbriae-associated gene expression by resveratrol in *Porphyromonas gingivalis* blocks biofilm production and increases in a directly proportional manner to the stilbene’s concentration [192]. The other stilbene that is able to impair biofilm production and disrupt mature biofilms is pterostilbene, which suppresses filamentation-related gene expression through the Ras/cAMP pathway [180].

Biofilms release planktonic cells or small clusters of pathogens through dispersion in a continuous manner [193]. Stilbenes interfere with biofilm propagation through targeting the enzymes involved, such as micrococcal nuclease, whose production is reduced following resveratrol administration, resulting in more efficient *Staphylococcus aureus* biofilm clearance [194].

### 3.4. Anticancer Effects: Preclinical and Clinical Evidence

In a recent epidemiological study, it was estimated that approximately 1,280,000 deaths in the European Union and 618 120 deaths in the United States were caused by cancer in 2025 [120,195]. Cancer is the second leading cause of death since the beginning of the 21st century, preceded by cardiovascular pathologies and followed by diabetes and chronic respiratory diseases [196]. The increase in patient care, therapy efficiency, and awareness of the impact of healthier lifestyles in cardiovascular diseases has caused a drop in its mortality rate, falling behind that of cancer [121,122,197].

Increasing attention has been given to chemoprevention as an alternative approach to cancer control. There is growing evidence that oxidative stress induced by ROS is linked to carcinogenesis at multiple stages [123]. ROS are abundant free radicals in cells and are associated with various degrees of tissue damage. Oxidative stress arises from an imbalance between ROS production and neutralization or elimination by the organisms’ naturally occurring antioxidant machinery. Consequently, oxidative stress can cause multiple issues, such as damage to proteins, lipids, and DNA, triggering or modulating the initiation, promotion, and progression of cancer [124].

Resveratrol has demonstrated potential anticancer activity, first reported by Jang et al., and has sparked significant interest in its chemopreventive and chemotherapeutic properties (Figure 3) [125]. Resveratrol has been tested in both in vitro and in vivo carcinogenesis assays for various cancer types, including leukemia, prostate, breast, lung, colon, ovary, liver, oral cavity, thyroid, and non-melanoma and melanoma skin cancer (Table 1) [126,198,199,200,201,202,203,204,205,206]. The chemopreventive properties of resveratrol rely on the compound’s antioxidant activity. All stages of carcinogenesis are affected by the stilbene’s anticancer activity, with the inhibition of COX-2 representing the major hallmark [125].

To date, three different isoforms of COX have been described, i.e., COX-1, which is expressed in normal tissues and participates in tissue homeostasis, COX-2, which results from overexpression in cases of inflammation or the development of neoplasms, and COX-3, a variant of COX-1 [207]. Many studies show that COX-2 plays a significant role in tumor progression, as its levels are elevated in premalignant and malignant tissues and are associated with a reduced survival rate in cancer patients, making it an unfavorable prognostic factor [116,208,209,210]. Clinical trials suggest that COX-2 inhibitors may be a solution for preventing the development adenomas and, potentially, carcinomas in the colon [207]. However, the clinical efficacy of these inhibitors is questionable due to increased cardiovascular risks [130].

Thus, resveratrol, which has shown positive effects in delaying cancer progression due to its significant potential in inhibiting COX-2, does not demonstrate toxicity effects [127]. Prostaglandins, produced through COX activity, have also been linked to cancer development and progression [116,211].

The increased production of prostaglandins influences the metabolism of carcinogens, tumor cell proliferation, and metastatic potential [131,212]. Thus, the inhibition of prostaglandin synthesis has been proposed as a strategy to prevent tumor development [131,132,211]. Numerous studies confirm that resveratrol has the capacity to inhibit COX-2, thereby reducing the synthesis of prostaglandin E2 (PGE2). Cianciulli et al. reported that resveratrol negatively regulates COX-2 and PGE2 in human intestinal Caco-2 cells treated with LPS, likely related to the inhibition of the NF-κB transcription factor [133]. NF-κB is associated with inflammatory, immune, and oncogenic responses [134].

Resveratrol activates AMPK, effectively preventing tumorigenesis [133,204,213,214]. Studies on redox status and antioxidant mechanisms’ function indicate that resveratrol acts as a potent chemoprotector in in vivo cancer models. In rats treated with the hepatotoxic carcinogen azoxymethane (AOM), which induces oxidative imbalance, resveratrol partially reversed effects such as lipid peroxidation, glutathione (GSH) depletion, and increased NO levels in the liver [215]. Additionally, resveratrol induces the expression of SOD and catalase through a mechanism involving the tumor suppressor gene phosphatase and TENsin homolog (PTEN) and protein kinase B (PKB) signaling pathways. PTEN-mediated inhibition of the phosphoinositide 3-kinase (PI3K)/PKB pathway leads to increased activity of SOD, GSH peroxidase, and catalase [216].

Studies showed that resveratrol induces apoptosis in several cancer types. This pro-apoptotic stimulation is related to changes in the cell cycle, caspase activation, and downregulation of XIAP, Survivin, Bcl-2, and Bcl-xL, and upregulation of Bax, Bak, Bim, Noxa, PUMA, p21, and TRAIL-R2/DR5 [143,144,145,217,218,219,220]. These effects are also correlated with p53 activation [143,144,218,220]. For instance, resveratrol and piceatannol increase cytoplasmic calcium concentrations in human breast cancer cells (MDA-MB-231), activating p53 and triggering pro-apoptotic gene transitions [145]. In prostate cancer cells with p53 mutations (Du145), resveratrol induces p53 phosphorylation, restoring wild-type-like DNA binding and promoting pro-apoptotic events [146,147,148].

Pterostilbene, a natural analog of resveratrol with greater bioavailability [101,146], shares significant similarity with resveratrol but exhibits stronger in vitro antioxidant activity and demonstrates substantial clinical potential across various diseases [149,150,221]. Pterostilbene shows chemopreventive properties against cancer in different in vitro and in vivo assays (Table 1). Studies have demonstrated that pterostilbene, which can act as an active apoptotic agent, also inhibits growth, adhesion, and metastasis development [118,152,153,154]. These effects have been observed in several types of cancer, including breast, lung, stomach, prostate, pancreatic, melanoma, and/or colon cancers, highlighting its role as a chemopreventive agent [152,155,156,157,158,159,217,221,222].

Rimando et al. analyzed the antioxidant activity of pterostilbene, finding that it inhibits preneoplastic lesion formation caused by carcinogenic agents in a rat mammary organ culture model [149]. Moreover, Chiou et al. demonstrated that pterostilbene is more potent than resveratrol in preventing AOM-induced colon tumorigenesis by activating the Nrf2-mediated antioxidant pathway [160].

In a similar experimental model, pterostilbene has shown to regulate the expression of inflammation-related genes such as COX-2 and iNOS [161,223]. Likewise, in immortalized human keratinocytes (HaCaT), pterostilbene enhances Nrf2 nuclear translocation and the expression of Nrf2-dependent oxidative stress-associated molecules, reinforcing the central role of Nrf2 in the chemoprevention promoted by pterostilbene [127]. In HT-29 colon cancer cells, pterostilbene inhibited cytokine induction through the p38 transcription factor pathway, as well as other anti-inflammatory pathways such as ERK, JAK-STAT, NF-κB, c-Jun NH2-terminal kinase, and PI3K [161]. This inhibition is attributed to the reduced expression of COX-2 and iNOS, suggesting that the MAPK cascade activation by p38 is an essential pathway for the anti-inflammatory action of pterostilbene [161].

Furthermore, pterostilbene is equally potent to resveratrol in inhibiting NF-κB, COX-2, iNOS, and activator protein 1 (AP-1) as demonstrated in a skin carcinogenesis model induced by tissue-type plasminogen activator (TPA) in mice [224]. Pterostilbene induces PTEN expression in prostate cancer, leading to decreased levels of miR-106b, miR-17, and miR-20a. The effect of pterostilbene on restoring PTEN mRNA and protein levels to normal values was greater than that of resveratrol, suggesting that pterostilbene exhibits greater in vivo activity due to the methoxy groups occupying the place of hydroxyl groups [225].

In a study involving a mice model of UVB-induced skin carcinogenesis, pterostilbene proved superior to resveratrol in skin damage of acute and chronic nature [127]. This study demonstrated that pterostilbene has anticarcinogenic effects, maintaining skin antioxidant defenses (e.g., GSH peroxidase, catalase, and superoxide dismutase activities and GSH levels) and reducing oxidative damage to DNA, proteins, and lipids induced by UVB radiation [127].

Several studies validate pterostilbene as an efficient anticancer therapeutical agent acting on multiple signaling pathways. In an rat model of AOM-induced colon carcinogenesis, a pterostilbene-enriched diet resulted in reduced aberrant crypt foci, decreased transcriptional activation of COX-2 and iNOS, inhibition of glycogen synthase kinase-3β (GSK-3β) phosphorylation, suppression of the Wnt/β-catenin signaling pathway, and downregulation of cyclin D1, vascular endothelial growth factor (VEGF), and matrix metalloproteinases (MMPs), and activation of Ras, PI3K/PKB, and epidermal growth factor receptor (EGFR) pathways [160]. Additionally, pterostilbene reduces pro-inflammatory cytokines such as IL-1β, IL-4, and TNF-α, as well as nuclear phospho-p65 levels [226].

McComarck et al. demonstrated that pterostilbene inhibits breast cancer cell proliferation stimulated by leptin through reduced JAK/STAT3 signaling [227]. In pancreatic cancer cells treated with pterostilbene, there was an upregulation of pro-apoptotic genes, changes in phosphorylated STAT3 levels, increased antioxidant activity by manganese superoxide dismutase (MnSOD), and enhanced expression of Smac/DIABLO and cytochrome c [152]. Additionally, Liu et al. proposed that pterostilbene inhibits JAK2/STAT3 signaling, reducing the expression of STAT3 target genes, including anti-apoptotic proteins Mcl-1 and Bcl-xL, while increasing proteins related to mitochondrial apoptosis (Bak, Bax, cleaved caspase-3, and cytosolic cytochrome c) and cyclin-dependent kinase inhibitors (e.g., p21 and p27) in osteosarcoma cells [228].

Pterostilbene acts as a chemopreventive agent, with effects extending beyond its anti-inflammatory and antioxidant properties or apoptosis-promoting effects. Studies have proposed that pterostilbene can induce cell death through autophagy [156,158,159,229,230]. Recently, it was observed that pterostilbene induces tumor autophagy through a mechanism dependent on lysosomal membrane permeabilization mediated by Hsp70 [217].

In recent years, the traditional model of cancer progression has been revised to emphasize the importance of tumoral heterogeneity in the development of chemo/radiotherapy resistance and post-treatment relapse. Cancer stem cells (CSCs) are attractive targets due to their self-renewal capacity, ability to generate heterogeneous tumor cell lineages, and high tumorigenicity [231,232,233]. Studies suggest that resveratrol and pterostilbene promote Argonaute-2 expression and activity, a central RNAi component, inhibiting cancer stem cell-like characteristics in breast cancer by increasing tumor-suppressor miRNAs such as miR-200c, miR-141, and miR-16 [234]. Pterostilbene suppresses CSC generation and metastatic potential in various experimental models, modulating epithelial–mesenchymal transition pathways and preventing the enrichment of CD133(+) CSCs in irradiated hepatoma [235,236].

Piceatannol, a hydroxylated analog of resveratrol, exhibits health benefits like resveratrol, though fewer studies are available (Table 1) [237,238,239]. Li et al. found that the anticancer properties of piceatannol may arise from its prooxidant effects due to the presence of copper (Cu)(II), which induces hydroxyl radical formation via Fenton and Haber-–Weiss reactions, leading to DNA damage [240,241]. Piceatannol inhibits hydrogen peroxide (H_2_O_2_)-induced NF-κB activation, ceramide, LPS, phorbol 12-myristate 13-acetate, and okadaic acid [242]. Additionally, it inhibits p65 phosphorylation and its nuclear translocation, and TNF-induced Iκ-Bα phosphorylation. Moreover, the hydroxyl groups at the 3′ and 4′ positions appear to play a crucial role in stilbene’s anticancer activity, as the same effects were observed for treatment with resveratrol treatment [242].

Piceatannol could reduce iNOS expression, thereby decreasing NO production. Furthermore, it inhibits COX-2 expression in RAW 264.7 cells stimulated with LPS and BV2 microglia cells [243,244]. Additionally, piceatannol increases protein levels of HO-1 in MCF10A, a human cell line of the mammary epithelium. The underlying mechanism involves the release of Nrf2 from Kelch-like ECH-associated protein 1 and its translocation to the nucleus, with consequent binding to the antioxidant response element, promoting HO-1 expression [245].

The effects of ε-viniferin on cancer prevention and treatment have been studied in over 20 cell models, including neural, breast, skin, liver, lung, bladder, stomach, colon, and hematological cancers. A published study demonstrated that, in approximately half of the total cases, the determined IC_50_ values for ε-viniferin were below 60 µM, highlighting the compound’s great potential in reducing the growth of cancer cells. Furthermore, comparative approaches on the anticancer activity of resveratrol and ε-viniferin have indicated that the latter is more active in one-third of the total cases [141].

In certain studies, the mechanisms underlying the cytotoxic and antiproliferative activities of ε-viniferin have been explored, with most being linked to apoptosis. This form of programmed cell death is essential for the development of the organism and tissue homeostasis. Apoptosis can be initiated by two main mechanisms: the intrinsic pathway, mediated by mitochondria, and the extrinsic pathway, mediated by death receptors. Most pro-apoptotic stimuli for the intrinsic pathway are associated with the permeabilization of the outer mitochondrial membrane. This process is regulated by a balance between pro- and anti-apoptotic members of the Bcl-2 family, leading to the release of cytochrome c into the cytosol. This apoptotic factor activates caspases and proteases responsible for the apoptotic phenotype [246].

ε-viniferin can induce apoptotic cell death in various tumor cell lines, including leukemia, myeloma, glioma, malignant melanoma, and hepatocellular carcinoma [247,248,249,250,251]. It induces apoptosis by disrupting normal mitochondrial transmembrane potential and activating pro-apoptotic proteases. Caspase-3 activation has been highlighted in leukemia and hepatocellular carcinoma cells [248,252]. Barjot et al. demonstrated that caspase-8 activation by ε-viniferin mediates a significant portion of this compound’s cytotoxic effect in U266 myeloma cell lines. In C6 glioma cells, activation of caspases-8, -9, and -3 has been observed [247,253].

The antiproliferative effect results in cell cycle arrest. ε-viniferin’s ability to halt the cell cycle has been investigated. Barjot et al. analyzed that treatment with ε-viniferin led to an accumulation of U266 myeloma cells in the G2-M phases and a reduction in cells in the G0/G1 phases [247]. Furthermore, ε-viniferin arrests the cell cycle of melanoma cells in the S phase and reduces the percentage of cells in the G1 phase, increasing regulatory proteins that control cell cycle progression through the S phase, such as cyclin E1 [249].

In addition, ε-viniferin, like other resveratrol oligomers, has been studied for its in vitro anticancer activities (Table 1). The cytotoxic activities vary depending on the tumor cell line and the structure of the resveratrol oligomer studied. Ito et al. investigated 18 oligomers ranging from dimers to octamers and tested them on five human tumor cell lines [254]. Seven of these compounds exhibited cytotoxic effects, primarily oligomers with more than four basic resveratrol units. However, other studies have suggested that the number of resveratrol units does not influence cytotoxic activity [255,256,257].

Although larger molecules are more active, their efficacy may be limited due to low bioavailability in vivo studies. To date, no in vivo trials have been conducted to compare ε-viniferin with resveratrol in studying its potential beneficial effects on the promotion, progression, or treatment of cancer. Several studies on ε-viniferin have not isolated the compound but instead used it in plant extracts or compound mixtures. These studies have shown that such mixtures or combinations are sometimes more active than the isolated molecules due to the synergistic effect between compounds [141].

For example, Vineatrol^®^ has demonstrated more efficient antiproliferative effects than isolated resveratrol or ε-viniferin in leukemia and hepatoma cells [248,258]. Additionally, ε-viniferin has been combined with anticancer drugs such as vincristine or cisplatin. Özdemir et al. demonstrated that ε-viniferin can enhance cell sensitivity to vincristine treatment in HepG2 cells [250]. Furthermore, the same author reported a strong apoptotic effect when combining cisplatin with ε-viniferin. Thus, ε-viniferin could potentially be used as a combined treatment with multiple anticancer drugs to reduce drug resistance and lower the required doses, thereby limiting side effects in living organisms [141].

Nivelle et al. achieved very positive results, as they observed reduced toxicity in normal fibroblasts compared to melanoma cancer cell lines [249]. Moreover, in non-transformed hepatocytes (HH4), higher concentrations of the stilbene dimer were required to induce toxicity compared to hepatocellular carcinoma cell lines such as HepG2 and Hep3B [259].

In conclusion, most studies have shown that ε-viniferin can reduce cancer cell growth, inducing apoptosis, and modifying the cell cycle [141]. Antioxidants act as agents that slow cancer progression by scavenging free radicals, inhibiting or preventing oxidative damage, and reducing oxidative stress [260].

**Table 1:** Overview of in vitro studies on the antitumor activity of representative stilbenes.

Stilbenes	Experimental In Vitro Model	Methodology	Experimental Conditions	Significant Results	Ref
Resveratrol	D407 cells	H_2_O_2_-induced cytotoxicity or MTT assay	0, 25, 50, and 100 µM for 24 h	Resveratrol offered protection to D407 retinal pigment epithelial cells against H_2_O_2_-induced cytotoxicity, resulting in reduced cytotoxicity.	[261]
	N2a cells	Fluorescein diacetate assay	1.5, 3.125, 6.25, 12.5, 25, 50, and 100 µM for 48 h	Plasma membrane integrity was compromised in resveratrol-treated N2a cells, reducing cell viability.	[262]
	T24 cells	Cell Proliferation Kit II	50, 100, 150, 200, and 250 µM for 24 h	Resveratrol exposure led to a decrease in cell viability, which was greater for s higher concentration of the stilbene. The determined IC_50_ was 178.73 µM.	[263]
	MCF-7 cells	MTS assay	20, 40, 60, 80, and 100 µM for 48 h	Resveratrol was cytotoxic in MCF-7 cells, aligning with the compound’s anticancer properties. Changes in membrane fluidity and, consequently, in cellular signaling pathways were detected.	[264]
	HRT cells	MTT assay	25, 50, 100, 200, and 300 µM for 72 h	Resveratrol inhibits HRT cell growth in a concentration-dependent manner.	[265]
	MCF-7 cells	Sulforhodamine B (SRB) assay	15 μg/mL for 24 h	Co-treatment with resveratrol and doxorubicin drastically lowered doxorubicin’s IC_50_ from 0.417 μg/mL to 0.035 μg/mL, showing it to be a good adjuvant in antitumor therapy.	[266]
Trans-resveratrol	PC-3 cells	MTS assay	0, 3, 10, 30, and 100 μM for 72 h	Trans-resveratrol exhibited an inhibitory effect on cell viability in PC3 cell line at concentrations ranging from 10 to 100 μM.	[267]
	MCF-7 cells JURKAT E.6, and THP-1	MTT or XTT assay	10, 30, 50, 70, 90, and 100 μM for 24, 48, 72, and 96 h	As trans-resveratrol concentrations increased, cell viability showed a greater or lesser pronounced decrease, depending on the cell line. Therefore, the effect of trans-resveratrol depends not only on dose and treatment duration but also on the cell type considered.	[268]
	MCF-7, Du145, and PC-3 cells	MTT assay	1 × 10^−15^–1×10^−3^ M for 48 h	Trans-resveratrol exhibited a cytotoxic effect on cancer cells at concentrations ranging from 1 × 10^−7^ to 1 × 10^−4^ M.	[269]
	MCF-7 cells	MTT or neutral red uptake (NRU) assay	1, 5, 10, 25, and 50 µM for 24 h	Solely an administration of trans-resveratrol did not lead to a decrease in cell viability in MCF-7 cell line. However, a pre-treatment with trans-resveratrol conferred protection against rotenone-induced toxicity.	[270]
	HepG2, Vero, and MCF-7 cells	MTT assay	0, 0.2, 0.4, 2, 4, 6.25, 12.5, 25, 50, and 100 µM for 24, 48, 72 h	Trans-resveratrol demonstrated cytotoxicity in all mentioned cell types at concentrations equal to or greater than 50 µM after 48 h.	[271]
	PC12 cells	MTT or NRU assay	0, 5, 10, and 25 µM for 24 h	Trans-resveratrol decreases the viability of P12 cells, as shown by both performed assays.	[272]
	HCT-116, HCT-116/p53(−/−), HepG2, and Hep3B cells	CellTiter-Blue^®^ and SRB assay	0, 1, 10, and 100 µM for 72 h	Trans-resveratrol demonstrated a significant ability to decrease cell viability at a concentration of 100 μM after 72 h.	[273]
	HepG2 cells	WST-1 assay	0.5–100 g/mL for 24, 36, and 48 h	Trans-resveratrol decreased the viability of HepG2 cells, evidencing the compound’s anticancer activity. The calculated IC_50_ was shown to decreased over time.	[273]
	MCF-10A, MCF-7, MDA-MB-231, and ZR-75-1 cells	CellTiter-Glo^®^ Luminescent Cell Viability assay	1–350 µM for 48 h	Trans-resveratrol was proven to be an efficient inhibitor of the cancer cell lines MCF-7, ZR-75-1, and MDA-MB-231, with IC_50_ values of 68.3 ± 2.6, 82.2 ± 4.8, and 67.6 ± 4.1 µM, respectively. Moreover, it was three times more potent in the MCF-10A cell line, with an IC_50_ of 20.0 ± 2.9 µM.	[274]
	SW480 cells	MTT assay	30 µM for 48, 72, and 96 h	There was a time- and dose-dependent decline in cell survival.	[275]
	MCF-7 cells	Annexin V-FITC and propidium iodide assay	6.25–50 µg/mL for 24 h	The viability of MCF-7 cells was suppressed following trans-resveratrol treatment.	[276]
	ARPE-19 cells, transmitochondrial normal RPE cybrid, and transmitochondrial AMD RPE cybrid cells	MTT assay	1000 µM for 48 h	Viability assays demonstrated that trans-resveratrol has beneficial properties for cybrid cells, increasing their viability compared to untreated cells.	[277]
	HepG2 cells	MTT assay	2.5, 10, 30, 50, 70, 100, 140, and 200 µM for 24, and 48 h	Trans-resveratrol has inhibitory effects on the cell viability of HepG2 cells, having greater impact in higher concentrations and prolonged exposure times.	[278]
	LTC-14 cells	MTT assay	0, 0.1, 1, 10, 100, and 1000 µM for 24, and 48 h	LTC-14 cells experienced a decrease in cell viability to below 50% in the presence of trans-resveratrol at a concentration of 200 µM.	[279]
	NCTC clone 929 cells	NRU assay	15.63, 31.25, 62.5, 125, and 250 μM for 24 h	Trans-resveratrol caused significant cell injury and death with an associated IC_50_ of 50 μM.	[280]
	A549 cells	MTT assay	0, 5.5, 11, 21.9, 32.9, 43.8, 87.6, 131.4, and 175.2 μM for 24 h	Trans-resveratrol treatment led to cell viability inhibition in a dose-dependent manner. The IC_50_ was determined to be 85.5 μM.	[281]
Pterostilbene	MCF-7, MDA-MD-231, and ZR-75-1	MTT assay	10 µmol/L, 20 µmol/L, 30 µmol/L (pterostilbene) + 5 µmol/L (Tamoxifen) for 24, 48, and 72 h	Combined therapy with pterostilbene and tamoxifen reduced cell viability in all cell lines. A greater decrease in viability was observed for the 24 h treatment.	[282]
	MDA-MB-231 and T-47D cells	MTT assay	10–100 µM for 48 h	A decrease in cell viability and significant morphological changes were observed in both cell lines following the treatment with pterostilbene. The IC_50_ concentrations for MDA-MB-231 and T-47D cells were 45.7 ± 0.01 and 63.1 ± 0.11 µM, respectively.	[283]
	MCF-7, SK-BR-3, and MDA-MB-468 cells	American Type Culture Collection	0–100 µM for 72 h	Treatment with pterostilbene arrested cells growth in a dose-dependent manner for all three cell lines, exhibiting a greater impact in MDA-MB-468 cells. The calculated IC_50_ values were 87.6 ± 9.0 µM for MCF-7 cells, 64.4 ± 4.6 µM for SK-BR-3 cells, and 45.7 ± 5.2 µM for MDA-MB-468 cells.	[284]
	HeLa, CaSki, and SiHa cells	MTT assay	0–200 µM for 72 h	All cell lines’ proliferation was inhibited by pterostilbene in a manner that varied in a directly proportional way to concentration. The IC_50_ for each cell line was calculated as follows: IC_50_ = 32.67 µM for HeLa, IC_50_ = 14.83 µM for CaSki, and IC_50_ = 34.17 µM for SiHa, indicating growth-inhibitory effects.	[285]
	TC-1 mouse cells after co-transformation with HPV16-E6, HPV16-E7, and c-Ha-Ras oncogenes	WST-1 assay	0–100 µM for 72 h	Pterostilbene exhibits significant cytotoxicity, evidenced by the formation of cytoplasmic blebs observed after 48 h. The number of apoptotic cells increased in a dose-dependent manner and the obtained IC_50_ of pterostilbene was 15.61 µM.	[286]
	CL187, C COLO 205, HCT-8, SW480, Lovo, and HCT-116 cells	Cell Counting Kit-8 (CCK-8) assay	1–100 µM for 24, 48, and 72 h	Pterostilbene inhibited cellular activity of all cell lines in a dose-dependent manner. After 72 h of treatment, the IC_50_ of pterostilbene for most of the cell lines used (except SW480 cells) was determined to be below 30 µM.	[287]
	HT-29 cells	SRB assay	5–100 µM for 48 h	A significant decrease in cell growth was only observed at concentrations equal to or greater than 10 µM. The extent of inhibitory effects was shown to be dependent on pterostilbene dosage.	[288]
	HT-29 cells	MTT assay	5 and 20 µM for 24 and 48 h	Treatment with pterostilbene at 20 μM inhibited the metabolic activity of HT-29 cells up to 80.2 ± 5.9%.	[289]
	HEC-1A and ECC-1 cells	MTS assay	0, 18.75, 37.5, 75, 150, and 300 µM for 48 h	Pterostilbene treatment significantly reduced cell viability in a dose-dependent manner, with associated IC_50_ values ranging between 72 and 78 µM for both cell lines.	[290]
	Kuramochi, Caov-3, OVCAR-4, OVCAR-8, and SKOV3 cells	MTT assay	0, 37.5, 75, 150, and 300 µM for 48 h	Cell viability was markedly reduced by pterostilbene in a dose-related way, with the IC_50_ for each cell line as follows: 161.2 μM for Kuramochi, 100.6 μM for Caov-3, 143.8 μM for OVCAR-4, 74.8 μM for OVCAR-8, and 95.2 μM for SKOV3 cells.	[291]
	LNCaP and PC3 cells	MTT assay	0, 20, 40, 60, 80, and 100 µM for 48 h	Pterostilbene reduced cell viability for both cell lines and in a dose-dependent manner. The IC_50_ values ranged between 70–80 μM for LNCaP cells, and 80–100 μM for PC3 cells.	[292]
	MIA PaCa-2 and PANC-1 cells	MTT assay	10–100 μM for 24, 48, and 72 h	Pterostilbene inhibited cell viability in a dose- and time-dependent manner in both cell lines. The IC_50_ concentration values varied depending on cell type and selected time points, with MIA PaCa-2 showing 72 μM at 24 h, 51 μM at 48 h, and 32 μM at 72 h, while PANC-1 showed 84 μM at 24 h, 33 μM at 48 h, and 29 μM at 72 h.	[293]
	A375, A549, HT29, and MCF-7 cells	Countess Automated Cell Counter and SRB Toxicology Assay	0–200 μM for 24, 48, and 72 h	Pterostilbene inhibited cell viability in a dose- and time-dependent manner in both cell lines. The IC_50_ values determined were cell type-dependent, being much lower for HT29 (IC_50_ = 60.3 mmol/L) and MCF7 (IC_50_ = 44.0 mmol/L) cells than for A375 (IC_50_ = 14.7 mmol/L) and A549 (IC_50_ = 28.6 mmol/L) cells.	[217]
	11–18, HCC827, HCC4006, H1975, and PC9 cells	MTT assay	0–150 μM for 72 h	Pterostilbene inhibited cell viability in all cell lines, with IC_50_ values ranging between 23.8 and 40.7 μM.	[294]
	HepG2 cells	MTT assay and CCK-8 assay	12.5–100 µM for 24 h	Cell viability and proliferation were reduced for all concentrations considered in a dose-dependent manner.	[295]
	HT29, MKN74, and CT26 cells	MTS assay	10, 50, and 100 µM for 48 h	Pterostilbene reduces cell viability in all three cell lines. The determined IC_50_ values were 21 µM for CT26, 63 µM for HT29, and 65 µM for MKN74 cells.	[296]
	CAR cells	MTT assay	5, 10, 25, 50, 75, and 100 µM for 24, 48, and 72 h	Pterostilbene induces cytotoxicity in a time- and dose-dependent manner. The IC_50_ values after 24, 48, and 72 h of incubation were 78.26 ± 4.33, 48.04 ± 3.68, and 20.65 ± 4.88 µM, respectively.	[297]
	MDA-MB-231 cells	MTT assay	1, 5, 20, 30, and 50 µg/mL for 48 h	Pterostilbene exhibits an inhibitory associated with an IC_50_ value of 79.5 ± 6.36 µg/mL.	[298]
	AsPC-1, BxPC-3, MIA PaCa-2, and PANC-1 cells	MTT assay	0, 50, 75, 100, 125, and 150 µM for 48 h	Increasing concentrations of pterostilbene reduced viability in all cell lines tested, pointing towards a dose-dependent sensitivity to the mentioned compound. The IC_50_ values ranged from 110 to 130 µM.	[299]
	MDA-MB-231 cells	MTT assay	2.5, 5, 10, 20, 40, and 80 µM for 24 h	A 24 h treatment with pterostilbene at 5 μM resulted in a 12% reduction in survival of MDA-MB-231 cells. The IC_50_, IC_80_, and IC_85_ doses against MDA-MB-231 cells were 30.4, 12.1, and 9.7 µM, respectively, confirming selective anticancer toxicity.	[300]
	GBC-SD, NOZ, and SGC-996 cells	CCK-8 assay	0–80 µmol/L for 48 h	Pterostilbene exhibits cytotoxic effects on all three cell lines. The estimated IC_50_ for GBC-SD cells was above 80 µmol/L, between 40–60 µmol/L for NOZ cells, and approximately 80 µmol/L for SGC-996 cells.	[301]
	C6, LN18, LN229, T98G, U87, and HUVECs cells	MTT assay	0, 20, 40, 80, and 100 µM for 24, 48, and 72 h	Pterostilbene inhibited cell viability on C6, LN18, LN229, T98G, and U87 cells. The IC_50_ values of pterostilbene treatment for 48 h were 30.10 μM for C6 cells, 22.30 μM for LN18 cells, 37.56 μM for LN229 cells, 32.93 μM for T98G cells, and 46.18 μM for U87 cells. Pterostilbene had a minimal impact on HUVEC cells compared to the previously mentioned cell lines.	[302]
Piceatannol	AGS, SK-MES-1, and J82 cells	MTT assay	0–100 µg/mL for 72 h	Besides enhancing gemcitabine’s cytotoxic and apoptotic effects, piceatannol actively inhibited SK-MES-1 cell viability. The synergistic combination increased the expression of the Bcl-2 pro-apoptotic protein family. IC_50_ concentrations for AGS, SK-MES-1, and J82 cells were 10.8 ± 0.7, 7.64 ± 0.5, and 6.7 ± 0.3 µg/mL, respectively.	[303]
	T24 and HT1376 cells	XTT assay	0.5, 2.5, 5, and 10 µM for 48 h	Piceatannol showed a dose-dependent inhibitory effect on the proliferation of both T24 and HT1376 cell lines. The IC_50_ values were 3.9 and 4.6 µM, respectively.	[304]
	HL-60 cells	MTT assay	10–200 µM for 24, 48, and 72 h	Piceatannol significantly inhibited HL-60 cell growth in a time- and dose-dependent manner. A moderate inhibition of HL-60 cells viability was observed after a 72 h treatment with piceatannol at 10, 20, and 50 µM. The highest inhibition was observed after 24, 48, and 72 h treatment with 100–200 µM concentration range.	[305]
	WM266-4 and A2058 cells	MTT assay	0, 1, 10, 20, 40, 100, and 200 µM for 36 h	Both cell lines exhibited decreased viability following piceatannol treatment. The calculated IC_50_ was 29.4 μM for WM266-4 cells, and 15.6 μM for A2058 cells.	[306]
	LNCaP, Du145, and PC3M cells	MTS assay	1, 5, 10, 25, 50, and 100 µM for 6 days.	All cell lines were susceptible to piceatannol treatment, exhibiting declining cellular activity. The IC_50_ values obtained were 31.7 μM for LNCaP, 23.2 µM for Du145, and 34.6 μM for PC3M cells.	[307]
	B16 cells	MTT assay	5–400 µM for 24 h	Piceatannol exhibited cytotoxicity effects, resulting in decreased cell viability. The obtained IC_50_ was 1.53 μM.	[308]
	U937 cells	MTT assay	0–100 µM for 24 h	Exposure to piceatannol inhibited cell viability, with an associated IC_50_ of 5 µM.	[309]
	NCI-H522 cells	WST-8 assay	10, 30, 50, 80, and 100 µM for 24, 48, and 72 h	Piceatannol treatment notably decreased NCI-H522 cell viability. The IC_50_ values at each timepoint were 53, 23, and 17 µM, respectively.	[310]
	Caco-2 and HCT-116 cells	Crystal violet assay	12.5, 25, 50, 100, and 200 µM for 24, 48, and 72 h	Piceatannol cytotoxic effects led to a decrease in cell viability fin both cell lines after a 72 h treatment. The obtained IC_50_ of piceatannol in Caco-2 and HCT-116 cells was 50 µM.	[311]
	L1210, K562, and HL-60 cells	Trypan blue dye exclusion	0–500 µM for 24 h	All cell lines were sensible to piceatannol’s cytotoxic effects. The calculated IC_50_ values of piceatannol were 50 µmol/L, <10 µmol/L, and <20 µmol/L for K562, HL-60, and L1210 cells, respectively.	[312]
	RAW 264.7 cells	MTT assay	0–50 µg/mL for 48 h	Piceatannol exhibits inhibitory activity, with an associated IC_50_ value of 5.7 µg/mL.	[313]
	HSG, HL-60 HSC-2, and HSC-3, (tumor cell lines) HPC, HGF, and HPLF (normal cells line),	MTT assay (HGF, HPC, HPLF, HSC-2, HSC-3, AND HSG) Trypan blue dye exclusion (HL-60)	10–1000 μM for 24 h	Piceatannol exhibits greater inhibitory effects on cancer cells compared to normal cells. The IC_50_ values for cancer cell lines were 63 µM for HSC-2, 232 µM for HSC-3, 373 µM for HSG, and 11 µM for HL-60 cells. In contrast, the IC_50_ values for normal cells were 367 µM for HGF, 414 µM for HPC, and >1000 µM for HPLF cells.	[314]
	SW1990 and PANC-1 cells	CCK-8 assay	1, 10, 20, 40, 100, and 200 μM for 72 h	Piceatannol inhibited up to 50% cell proliferation for both cell lines. The IC_50_ value for SW1990 cells was 30.69 µM, while for PANC-1 cells it was 21.82 µM.	[315]
	MOLT-4 cells	NRU assay	0.05, 15, 25, 50, and 100 μM for 48 h	Piceatannol reduced cellular viability with a calculated IC_50_ of 45.5 µM.	[316]
	HeLa cells	MTT assay	0–250 µM for 48 h	Piceatannol decreased cell viability and the associated IC_50_ value was 375.20 µM.	[317]
	Mouse embryonic stem cells (ESCs)	MTT assay	1–20 µM for 72 h	High concentrations of piceatannol exhibited cytotoxicity. The obtained IC_50_ value was 13.5 µM.	[318]
	C6 cells (proliferating and growth arrested)	Lowry method	1–100 µM for 72 h in proliferating cells and 24 h in growth-arrested cells	Piceatannol exhibits cytotoxic effects on both growth-arrested and proliferating cells. The IC_50_ concentration for growth-arrested cells was 20 ± 2 µM, while for proliferating cells it was 28 ± 4 µM.	[319]
	10ScNCr/23, A-431, RAW 264.7, and CCR-CEM cells	Trypan blue dye exclusion	0–50 µM for 24 h	Piceatannol exhibits inhibitory effects on all cell lines. The IC_50_ concentration in RAW 264.7 cells were 1.30 ± 0.12 µM.	[320]
	THP-1 cells	Light microscopy	10, 20, 30, 40, and 50 µM for 48 h	Significant cytotoxic effects with noticeable cell shrinkage were observed at concentrations above 30 µM.	[321]
(-)-ε-viniferin	HSC-2, HSC-3, HCF, HPC, HPLF, HSG, and HL-60 cells	MTT assay in adherent cells Trypan blue dye exclusion in non-adherent cells	0–1000 µM for 24 h	The four tumor cell lines (HSC-2, HSC-3, HSG, and HL-60) were more sensitive to (-)-ε-viniferin than the remaining normal cell lines. The IC_50_ values were 42 µM for HSC-2 cells, 84 µM for HSC-3 cells, 111 µM for HCF cells, 146 µM for HPC cells, 94 µM for HPLF cells, 110 µM for HSG cells, and 31 µM for HL-60 cells.	[314]
	P-388 cells	MTT assay	0–100 µM for 48 h	ε-viniferin moderately inhibited cell viability in comparison to hopeaphenol, which exhibited a greater effect. The IC_50_ measured at 18.1 ± 0.7 µM.	[255]
	HepG2 and Chang cells	MTT assay	1.56–200 µg/mL for 72 h	No cytotoxic effect was detected in either cell lines.	[322]
(+)-ε-viniferin	RAW 264.7 cells	MTT assay	1, 5, and 10 µM for 12 h	Cell viability was significantly reduced to 60% after exposure of 10 µM. IC_50_ was not determined.	[323]
trans-ε-viniferin	K562, L1210, and HCT116 cells	MTT assay	0–50 µM for 48 h	No cytotoxicity was detected. The IC_50_ was assumed to be above 50 µM.	[324]
	AGS, MRC-5, SK-MES-1, and J82 cells	MTT assay	0–100 µg/mL for 72 h	Cytotoxicity was observed for all cell lines tested. The IC_50_ values were 42.6 ± 1.7 µM for AGS cells, 49.9 ± 3 µM for MRC-5 cells, 78.8 ± 3.3 µM in SK-MES-1 cells, for 56.7 ± 1.2 µM in J82 cells.	[303]
	Mouse primary astrocytes and neurons co-culture	CellTitel 96 ^®^ Aqueous assay	1, 5, 10, 20, 50, and 100 µM for 24 h	Cell viability was significantly reduced when cells were exposed to concentrations of 50 and 100 µM.	[325]
	AGS, COLO 205, HepG2, HL-60, and HT-29 cells	MTT assay	0–100 µg/mL for 48 h	Dose-dependent cytotoxicity was reported, with a greater effect observed in HL-60 cells. The determined IC_50_ values were: 9.3 ± 0.3 µM in AGS cells, 85.5 ± 8.1 µM in COLO 205 cells, 7.7 ± 0.2 µM in HepG2 cells, 5.6 ± 1.4 µM in HL-60 cells, and 13.9 ± 0.1 µM in HT-29 cells.	[326]
	Hep3B, HepG2, and HH4 cells	Crystal violet assay	0–200 µM for 24, 48, and 72 h	It was more cytotoxic to Hep3B cells and reduced cell quantity in a dose- and time-dependent manner. Higher amounts were required to cause toxicity in HH4 cells. The IC_50_ values obtained were the following: - Hep3B cells: 108.1 ± 31.8 µM (24 h), 73.9 ± 17.3 µM (48 h), 63.1 ± 10.8 µM (72 h). - HepG2 cells: 140 ± 39.7 µM (24 h), 103.8 ± 19.2 µM (48 h), 94.8 ± 28.3 µM (72 h). - HH4 cells: >200 µM (24 h), 192.7 ± 21.1 µM (48 h), 177.9 ± 20.5 µM (72 h).	[326]
	HepG2 and Caco-2 cells	MTS assay, NRU, and protein content	0–100 µg/mL for 24 and 48 h	For every endpohint examined, both cell lines showed a time-dependent decline in cell viability. The IC_50_ values were: - HepG2: 28.28 ± 2.15 µg/mL 24 h and 17.85 ± 3.03 µg/mL for 48 h. - Caco-2 cells: 36.72 ± 3.01 µg/mL for 24 h and 20.63 ± 1.25 µg/mL 48 h.	[327]
trans-ε-viniferin and cis-ε-viniferin	HeLa, MCF-7, C6, HepG2, and HT-29 cells	MTT assay	0–100 μM for 70 h	Cis- and trans-ε-viniferin to all cell lines, although greater significance was registered for C6 and HeLa cells. The IC_50_ values for trans-ε-viniferin were: - 20.4 µM in HeLa cells, 44.8 µM in MCF-7 cells, 18.4 µM in C6 cells, 74.3 µM in HepG2 cells, and 88.4 µM in HT-29 cells. The IC_50_ values for cis-ε-viniferin were: - 21.5 µM in HeLa cells, and 47.2 µM in MCF-7 cells, 20.1 µM in C6 cells, 76.2 µM in HepG2 cells, and 90.2 µM in HT-29 cells.	[328]
ε-viniferin	WSU-CLL cells	Trypan blue dye exclusion	0–100 µM for 24, 48, and 72 h	A concentration- and time-dependent decrease in cell viability was observed, with resveratrol overperforming ε-viniferin. Inhibited cell proliferation was accompanied by a reduction in DNA synthesis. The IC_50_ value determined at 72 h was 60 µM.	[248]
	HL-60 cells	MTT assay	10–200 µM for 24 h	Cell viability decreased in a concentration-dependent manner. The IC_50_ was 33 µM.	[329]
	HepG2 cells	Trypan blue dye exclusion	30 µM for 24, 48, and 72 h. 1, 5, 10, 30, 60, and 100 µM for 48 h	At 60 µM, ε-viniferin completely blocks cell proliferation. After 48 h, the toxicity potential of ε-viniferin was lower than resveratrol. The IC_50_ for 48 h was 58.4 µM.	[258]
	SW480 cells	Trypan blue dye exclusion and MTT assay	30 µM for 24, 48, 72, and 96 h in trypan blue dye exclusion. 3, 30, 60, and 100 µM for 48 h in coulter counter	Cells exposed to ε-viniferin grew similarly to the control group, with a reduced growth rate and increasing percentage of cell inhibition. In the MTT assay, no significant inhibition of cell proliferation was recorded.	[275]
	VSMCs cells	MTS assay	10, 20, and 30 µM for 48 h	The potential for arresting cell proliferation rate of ε-viniferin at 20 µM was significantly higher than resveratrol’s at 20 and 30 µM.	[330]
	SK-MEL-25 and HT-144 cells	MTT assay Trypan blue dye exclusion	25–200 µM for 24, 48, and 72 h	Both melanoma lines showed time- and dose-dependent reduction in survival. The IC_50_ for 48 h was 60 µM.	[249]
	C6 cells	WST-1 assay	95 and 130 µM 12, 24, and 48 h	Proliferation decreased at all doses and times tested in C6 cells.	[253]
	Caco-2 cells	MTT and NRU assays	1.56, 3.12, 6.25, 12.5, 25, 50, and 100 µM for 24 h	At and above 25 µM, cell viability in Caco-2 cells decreased. ε-viniferin was slightly more effective than resveratrol.	[331]
	Vascular endothelial cells (VECs)	H_2_O_2_-induced cytotoxicity	10, 20, and 30 µM for 24 h	ε-viniferin effectively protected cells from cytotoxic effects of H_2_O_2_. A 24 h pre-treatment with ε-viniferin reduced intracellular ROS.	[332]
	VECs	H_2_O_2_-induced cytotoxicity	5 and 10 µM for 24 h	At 10 μM, a pre-treatment with ε-viniferin conferred VEC with resistance against H_2_O_2_-induced oxidative stress.	[333]
	A2058, A549, HOS, U2OS, and MCF-10A cells	MTT assay	1–15 μM for 24, 48, and 72 h	ε-viniferin exhibited a time- and dose-dependent decrease in the viability of HOS, U2OS, and A549 cells, but not in A2058 cells.	[334]
ε-viniferin glucoside	PC12 cells	MTT assay	0–10 µM for 24 h	Cell viability was not significantly altered following the exposure to the stilbene.	[335]

### 3.5. Cardiovascular and Metabolic Benefits

Stilbenes act on various factors involved in the bioavailability of NO, such as arginase, dimethylargininase (DDAH), and asymmetric dimethylarginine (ADMA), thereby modulating NO production during oxidative stress [163,336,337,338]. Also, stilbenes influence metabolic pathways like adipogenesis, lipogenesis, lipolysis, thermogenesis, and fatty acid oxidation. They regulate PPARγ, sterol regulatory element-binding protein 1c (SREBP-1c), uncoupling (UCPs), sirtuin 1 (SIRT1), lipoprotein lipase (LPL), fatty acid synthase (FAS), and acetyl-CoA carboxylase (ACC). Piceatannol and pterostilbene modulate lipid metabolism via ACC, SREBP1, PPAR-γ, and PPAR-α [72]. Pterostilbene demonstrates greater in vivo efficiency than resveratrol due to its higher bioavailability and greater capacity to bind three amino acids of PPARα versus two for resveratrol [339]. Resveratrol has vasodilatory properties, enhancing endothelial NO production and increasing the expression of eNOS in rat arteries while decreasing the expression of NADPH oxidase [340,341]. Studies show that resveratrol activates membrane-associated structures, such as estrogen receptors, which trigger an intracellular signaling cascade targeting the AMPK pathway. This activation can phosphorylate eNOS at serine 1177 [342]. Additionally, resveratrol activates SIRT1, reducing acetylation and leading to eNOS activation. Resveratrol improves SIRT1-dependent NO bioavailability induced by insulin in HUVEC cells cultured under high glucose conditions for 48 h [343]. Another study shows that resveratrol suppresses SIRT1 inhibition, reducing eNOS acetylation in HUVEC cells exposed to H_2_O_2_-induced oxidative stress [344].

Furthermore, resveratrol increases NO production in blood platelets via the PI3K/protein kinase B (Akt) pathway, activates vasodilator-stimulated phosphoprotein, and inhibits p38MAPK. This leads to reduced platelet activation and ROS production [345]. Resveratrol also dose-dependently increases RNA synthesis of VEGF and eNOS and reduces endothelin secretion in HUVEC cells, lowering blood pressure and improving vasodilation [346]. In human coronary smooth muscle cells incubated with resveratrol, cGMP synthesis increased, activating cGMP-dependent protein kinase, which in turn activated potassium channels, leading to hyperpolarization and relaxation of vascular smooth muscle cells [347].

In vivo studies in rats with accelerated atherosclerosis and metabolic diseases showed improved endothelial dysfunction [57]. In another study using obese Zucker rats, a model for metabolic syndrome, chronic resveratrol administration increased eNOS expression and reduced dyslipidemia and hypertension [348]. Similarly, in hypercholesterolemic rabbits treated with resveratrol, plasma NO concentration increased, and endothelial dysfunction was reduced [349]. However, in estrogen receptor-deficient mice, resveratrol showed no vasorelaxation effects compared to normal mice, demonstrating that estrogen receptors mediate the intracellular effects of resveratrol. Activation of these receptors leads to phosphorylation of Src, Erk ½, and eNOS, resulting in NO synthesis activation [342].

Stilbenes, such as resveratrol found in grapes, need further testing in humans. In a 12-week study where 75 mg/day of resveratrol was administrated to 15 healthy non-obese postmenopausal women, no changes were detected in metabolic functions, such as AMPK signaling, inflammatory markers, insulin, and mitochondrial functions [350]. The absence of effects in humans aligns with previous results in healthy rodents [351], suggesting that resveratrol’s effects are limited to conditions like type II diabetes, dyslipidemia, and obesity [117].

A study on resveratrol’s role in primary and secondary cardiovascular disease prevention involved a small sample size (n = 75) over one year, limiting clinical relevance. Participants were divided into three groups: one received grape extract with 8.1 mg/day of resveratrol for six months followed by an increase in the stilbene’s concentration to 16.2 mg/day for another six months, a second group received grape extract without resveratrol, and the third received a placebo. All participants were on statins and treated per cardiovascular disease prevention guidelines [352]. Results were promising, with the resveratrol-treated group showing improved inflammatory and fibrinolytic profiles in comparison to the remaining groups [352]. Resveratrol provided additional benefits for patients at high risk of cardiovascular disease beyond other phenolic compounds in grape extract, complementing the current primary prevention guidelines [353].

In animal models, resveratrol and pterostilbene demonstrated anti-hyperlipidemic effects, but clinical trials in humans showed no significant changes in low-density lipoprotein (LDL)/high-density lipoprotein (HDL) ratios. A meta-analysis of randomized clinical trials revealed that the use of resveratrol as a supplement did not induce significant alterations in lipid parameters, including LDL, HDL, total cholesterol, and triglycerides [354]. Pterostilbene at high and low doses promotes an increase in LDL without affecting HDL or triglycerides in a placebo-controlled trial [355]. Similarly, grape extract administration did not influence LDL levels [117]. However, in a secondary prevention study of cardiovascular diseases, grape extract with resveratrol (8.1 mg/day for six months, then 16.2 mg/day) in patients with stable coronary artery disease and dietary restrictions showed increased anti-inflammatory adiponectin and reduced plasminogen activator inhibitor-1 (PAI-1) [356].

These findings suggest that resveratrol has cardioprotective effects, improving anti-inflammatory responses and preventing atherothrombotic signaling (Figure 3) [356].

Preclinical data have demonstrated that resveratrol can be utilized in diabetes management through correction of insulin signaling defects, improvement in insulin resistance, and prevention of pancreatic beta cells’ dysfunction [357]. Moreover, resveratrol prevents hyperglycemia in animal models for diabetic pathology by promoting glucose uptake and GLUT4 translocation to the caveolar membrane within the diabetic myocardium [358]. It also improves glucose tolerance and reduces the expression of advanced glycation endproduct (AGE) receptors in the liver and kidneys of diabetic rats [359].

Resveratrol exhibits actions that inhibit reactive oxygen and nitrogen species, such as superoxide anion (O_2_^•−^), hydroxyl radical (OH^•^), H_2_O_2_, and malondialdehyde (MDA), while increasing levels of antioxidant enzymes like SOD, catalase, and glutathione peroxidase in diabetic animals [360]. It also inhibits pro-inflammatory signaling through NF-κB and reduces the production of inflammatory cytokines, including IL-1β, IL-4, and IL-6 and TNF-α, [360]. Additionally, resveratrol increases insulin sensitivity, glucose tolerance, and mitochondrial biogenesis via AMPK-dependent pathways [361]. A study showed that resveratrol did not achieve the same effects in AMPK-deficient mice, highlighting the essential role of this protein in the metabolic actions of resveratrol [361].

Resveratrol’s role in glycemic control remains slightly uncertain, as some human studies lack significant evidence of the stilbene’s effect on metabolic dysfunctions. Daily co-administration of metformin or glibenclamide and 250 mg of resveratrol for three months improves glycemic parameters in patients with type 2 diabetes, when compared with those receiving therapies lacking the stilbene [362]. As reported by Movahed and colleagues, 1 g/day of resveratrol for 45 days reduced systolic blood pressure, fasting glucose serum levels, and HbA1c [363]. Furthermore, a much lower daily 5 mg dose of resveratrol was found to reduce systolic blood pressure, HbA1c levels, and improve insulin sensitivity, while not affecting the homeostatic model assessment of insulin resistance, in a 28-day long treatment [363].

In contrast, a randomized clinical trial by Thazhath et al., with 500 mg of resveratrol twice daily for five weeks in diet-controlled type 2 diabetes patients, showed no significant effects on glycemic control [364]. No differences were observed in fasting glucose, postprandial glucose, HbA1c, gastric emptying, or glucagon-like peptide-1 (GLP-1) secretion between the resveratrol and placebo groups. Similarly, a six-month treatment did not show metabolic improvements in type 2 diabetes patients [365]. Therefore, the effects of resveratrol on human diabetes remain not fully understood [117].

Pterostilbene has shown promising results in glycemic control in obese rats with insulin resistance, enhancing hepatic glucokinase activity and glucose uptake in skeletal muscle [366]. In vitro, studies indicate that pterostilbene protects pancreatic beta cells from oxidative stress and apoptosis [367]. Pterostilbene, along with other constituents of *Pterocarpus marsupium*, has demonstrated anti-hyperglycemic properties [368,369]. However, human data on pterostilbene are still limited. One study administered sea buckthorn and blueberry extract to children with type 1 diabetes for two months, resulting in elevated SOD and glutathione peroxidase levels and reduced HbA1c levels [370].

Piceatannol also has glucose-regulating capabilities, increasing glucose disposal. Minakawa et al. observed that piceatannol promotes glucose uptake in cultured myotubes in a dose-dependent manner by activating AMPK and inducing glucose transporter type 4 (GLUT4) translocation [371]. AMPK activators are promising for type 2 diabetes treatment since AMPK stimulates GLUT4 translocation, with skeletal muscle being the primary site of glucose clearance [372,373]. Piceatannol was shown to lower fasting glucose in vivo by activating AMPK in myotubes and minimally impacting insulin secretion in vivo, establishing it as a glucose modulator [371,374,375]. Insulin signaling is modified by piceatannol in different tissues, for instance, it blocked insulin action in adipocytes but improved endothelial function under inflammatory stress [376,377,378].

### 3.6. Neuroprotective Effects and Cognitive Function

In neurodegenerative diseases (NDs), there is progressive damage to the structure and function of neurons. The three main NDs are Alzheimer’s disease, Parkinson’s disease, and amyotrophic lateral sclerosis. Each presents different clinical signs; however, the pathological processes appear similar, suggesting common biological pathways in the onset and progression of NDs. Factors involved in NDs include aging, lifestyle, and genetics [379]. Generally, neuroinflammation and oxidative stress are the primary causes of neuronal dysfunction and death, along with excitotoxicity, mitochondrial dysfunction, and apoptosis [380]. Oxidative stress in neurodegeneration is correlated with the progression of Parkinson’s and Alzheimer’s diseases [381]. One hypothesis involves the use of natural products due to their centuries-long application in human diseases [382,383,384]. In recent decades, many studies have described the protective effects of natural polyphenols and their active derivatives against various diseases, including cardiovascular diseases, diabetes, cancers, and NDs [385]. Additionally, natural compounds demonstrate great potential as neuroprotective agents for treating NDs due to their inherently multi-target profiles [386,387].

The neuroprotective effects of stilbenes include antioxidants and anti-inflammatory properties (Figure 3) [388,389,390,391,392]. Resveratrol protects neurons from ROS and improves motor coordination in 1-methyl-4-phenyl-1,2,3,6-tetrahydropyridine-induced parkinsonism in rats by neutralizing hydroxyl radicals [388]. Furthermore, resveratrol improves mitochondrial function, motor coordination, and neuronal survival, inhibits β-secretase, enhances non-amyloidogenic amyloid precursor protein (APP) cleavage, and increases clearance of beta-amyloid (Aβ) peptides [393]. Moreover, resveratrol protects against lipopolysaccharide (LPS)-induced dopaminergic neurodegeneration by inhibiting microglial activation and NF-κB signaling [394]. In Alzheimer’s disease, resveratrol shows therapeutic potential by reducing amyloid plaques in the brain. Marambaud et al. demonstrated that resveratrol may not inhibit Aβ production but promotes proteasome-dependent Aβ degradation [395]. Winemaking residues are a source of piceatannol and vitisinol C, which inhibits Aβ aggregation [396,397].

Pre-treatment with resveratrol in rats subjected to cerebral ischemia reperfusion injury increased Nrf2 and HO-1 levels, reducing oxidative damage during ischemic events within the brain [398]. This pre-treatment also results in improved neurological scores with associated reductions in brain water content and infarct volume. In models of global cerebral ischemia, resveratrol blocked neuronal death by activating PI3K/Akt signaling, downregulating GSK-3β, and regulating CREB (cAMP response element-binding protein) [399].

Resveratrol improves cognition in animal models of vascular dementia, showing effects on cerebral ischemia [400]. In this study, vascular dementia was induced by bilateral common carotid artery occlusion for 8–12 weeks. Resveratrol treatment improved learning and memory scores by reducing malondialdehyde, a lipid peroxidation product, and increasing antioxidant enzyme levels, such as glutathione and SOD, in the cerebral cortex and hippocampus [117,400].

Pterostilbene acts as a neuroprotectant by counteracting glucose-induced damage in neuroblastoma cells, preventing the decline in viable cells and ROS generation in a concentration-dependent manner [389]. It also increased mitochondrial cytochrome C, mitochondrial complex I and III activities, and membrane potential, along with Nrf2, HO-1, and GST levels, offering protection against neuronal oxidative stress [389]. Pterostilbene improves memory by increasing RE1-silencing transcription factor (REST), postsynaptic density protein 95 (PSD-95), and mitochondrial porin 1 in the dentate gyrus of aged rats. Resveratrol improves memory in healthy and diabetic individuals with subclinical impairment but not in full Alzheimer’s cases [393].

Oxyresveratrol has shown neuroprotective effects. Studies in rat cortical neurons demonstrated that it prevented Aβ (25–35)-induced damage by reducing cytosolic Ca^2+^ levels, inhibiting glutamate release, and lowering ROS generation [401]. Another study by Andrabi et al. demonstrated that, in a rat model of transient middle cerebral artery occlusion, oxyresveratrol significantly reduced cerebral infarct volume and improved subsequent neurological deficits through inhibition of cytochrome C release and caspase-3 activation [402].

In a 52-week long randomized placebo-controlled clinical trial, doses of resveratrol raging between 500 and 1000 mg administered twice daily were well tolerated by Alzheimer’s patients, reducing Aβ40 levels in the cerebrospinal fluid and plasma, although other Alzheimer’s biomarkers were unaffected [403]. Another trial with a daily supplementation of 200 mg of resveratrol and 320 mg of quercetin resulted in an improvement in memory performance when co-administrated in overweight elderly individuals [404].

A recent study showed that a double daily administration of 75 mg of resveratrol, for a period of 14 weeks, improved cerebrovascular function, cognition, and mood in postmenopausal women. Additionally, it enhanced cognitive performance and cerebral blood flow in individuals with type 2 diabetes [405,406]. In conclusion, human studies have demonstrated the beneficial effects of resveratrol in improving memory and cognition in healthy individuals and those with diabetes-related cognitive impairment, but not in Alzheimer’s patients [117].

## 4. Conclusions and Future Perspectives

The wine industry represents a significant opportunity for obtaining high-value byproducts with potential industrial applications. Vineyard waste is a rich source of compounds with antioxidant effects (specifically stilbenes), which can act as potent reactive free radical scavengers, enzyme activators and/or inhibitors, antibacterial agents, anti-inflammatory agents, and anticancer agents, among other health-beneficial agents. For example, stilbenes inhibit platelet aggregation, possibly through the inhibition of COX-1. They also reduce markers of oxidative stress and inflammation, such as TNF-α and IL-1β, in various models of ischemia reperfusion injury, and inhibit cardiomyocyte hypertrophy through the activation of AMPK. In addition, stilbenes exert beneficial effects in cardiovascular conditions, such as improving glucose tolerance and insulin resistance in animal models of diabetes. Stilbenes exhibit anticancer effects through the inhibition of cancer cell proliferation, oxidative stress, and inflammation, engaging in regulatory roles of cell death mechanisms. From a neurological perspective, stilbenes can upregulate Nrf2 and HO-1 expression to mitigate oxidative damage during cerebral ischemia. Thus, their neuroprotective effects are largely attributed to their antioxidant activity. Stilbenes also exhibit a broad range of functions, including microbicidal and antifungal activities, various effects on the reversal of drug resistance, and biofilms and virulence factors, highlighting the potential of stilbenes as antimicrobial agents. Although the results are promising, most of the evidence comes from in vitro and in vivo studies, with limited clinical trials available to confirm the beneficial effects of stilbenes in humans.

From an industrial perspective, stilbenes have attracted growing interest not only in the pharmaceutical sector but also in the food and cosmetics industries. Their incorporation into biodegradable packaging, natural cosmetics, and functional supplements highlights their potential as multifunctional agents, aligning with the increasing demand for sustainable and bioactive products.

However, to allow large-scale applications, significant advancements are still required in extraction, purification, and formulation technologies. Low water solubility, limited bioavailability, and rapid metabolism remain technical challenges that must be addressed, potentially through innovative strategies such as nanoencapsulation, cyclodextrin complexation, or incorporation into polymeric matrices.

Future prospects for the utilization of stilbenes include the following: the optimization of extraction methods for improved efficiency and selectivity and reduced environmental impact including decreased energy and time consumption, the preservation of stilbene integrity, and the application of green chemistry principles; the development of controlled-release systems for pharmaceutical and cosmetic applications, including the formulation of nanoparticles with stilbenes to enhance bioabsorption and bioavailability for drug delivery; comprehensive toxicological and regulatory evaluations to ensure the safety of stilbene use in consumer products; the exploration of lesser-known stilbenes beyond resveratrol to broaden the range of applications and therapeutic potential, including resveratrol derivatives such as pterostilbene, pinosylvin, ε-viniferin, piceatannol, rhapontigenin, and isorhapontigenin, among others; and integration into circular production chains, contributing to the sustainability of the wine sector and fostering the bioeconomy, thereby fostering eco-design, one of the seven pillars of the circular economy.

In conclusion, stilbenes demonstrate great potential for multiple human health benefits. However, further studies are needed due to the number and complexity of the cellular processes involved. Additionally, because of their low concentrations in foods and associated rapid metabolism and excretion in mammalian organisms, improvements in stability, solubility, and delivery systems are required to enable clinical application. Moreover, there is limited information with regard to the potential beneficial properties of less-studied stilbenes. Therefore, more research is still necessary to uncover the impact of lesser-known stilbenes on health.

## Abbreviations

The following abbreviations are used in this manuscript:

UV	Ultraviolet
STS	Stilbene synthase
DW	Dry weight
MAE	Microwave-assisted extraction
UAE	Ultrasound-assisted extraction
PSE	Pressurized solvent extraction
COX	Cyclooxygenase
Aβ	Beta-amyloid
ROS	Reactive oxygen species
RNS	Reactive nitrogen species
SOD	Superoxide dismutase
NADPH	Nicotinamide adenine dinucleotide phosphate
GPx	Glutathione peroxidase
GST	Glutathione-S-transferase
NF-κB	Nuclear factor κB
eNOS	Endothelial nitric oxide synthase
LDL	Low-density lipoprotein
SIRT1	Sirtuin 1
H_2_O_2_	Hydrogen peroxide
TNF-α	Tumor necrosis factor-α
AMPK	Adenosine monophosphate-activated protein kinase
LPS	Lipopolysaccharide
HO-1	Heme oxygenase-1
Nrf2	Nuclear factor erythroid 2-related factor 2
STAT3	Signal transducer and activator of transcription 3
NRU	Neutral red uptake
BH4	Tetrahydrobiopterin
cAMP	Cyclic adenosine monophosphate
cGMP	Cyclic guanosine monophosphate
NDs	Neurodegenerative diseases
CCR1	Chemokine receptor
PGE2	Prostaglandin E2
GSH	Glutathione
PTEN	Phosphatase and TENsin homolog
PKB	Protein kinase B
GSK-3β	Glycogen synthase kinase-3β
VEGF	Vascular endothelial growth factor
CSC	Cancer stem cell
VECs	Vascular endothelial cells
PI3K	Phosphoinositide 3-kinase
Akt	Protein kinase B
HDL	High-density lipoprotein
